# Evaluation of the safety, immunogenicity and efficacy of a new live-attenuated lumpy skin disease vaccine in India

**DOI:** 10.1080/21505594.2023.2190647

**Published:** 2023-03-19

**Authors:** Naveen Kumar, Sanjay Barua, Ram Kumar, Nitin Khandelwal, Amit Kumar, Assim Verma, Lokender Singh, Bhagraj Godara, Yogesh Chander, Garvit Kumar, Thachamvally Riyesh, Deepak Kumar Sharma, Anubha Pathak, Sanjay Kumar, Ramesh Kumar Dedar, Vishal Mehta, Mitesh Gaur, Bhupendra Bhardwaj, Vithilesh Vyas, Sarjeet Chaudhary, Vijaypal Yadav, Adrish Bhati, Rakesh Kaul, Arif Bashir, Anjum Andrabi, Raja Wasim Yousuf, Abhimanyu Koul, Subhash Kachhawaha, Amol Gurav, Siddharth Gautam, Hari Audh Tiwari, Vijay Kumar Munjal, Madhurendu K. Gupta, Rajender Kumar, Baldev R. Gulati, Jyoti Misri, Ashok Kumar, Ashok Kumar Mohanty, Sukdeb Nandi, Karam Pal Singh, Yash Pal, Triveni Dutt, Bhupendra N. Tripathi

**Affiliations:** a National Centre for Veterinary Type Cultures, ICAR-National Research Centre on Equines, Hisar, India; b Indian Veterinary Research Institute, Mukteswar, India; c Department of Veterinary Microbiology, College of Veterinary and Animal Science, Udaipur, India; d Department of Animal Husbandry, Banswara, Rajasthan, India; e Department of Veterinary Gynaecology and Obstetrics, College of Veterinary and Animal Science, Udaipur, India; f Department of Animal Husbandry, Udaipur, Rajasthan, India; g Department of Animal Husbandry, Jodhpur, Rajasthan, India; h Department of Animal Husbandry, Alwar, Rajasthan, India; i Livestock Research station, Nohar, Rajasthan, India; j Animal Husbandry Department, Jammu and Kashmir, India; k Krishi Vigyan Kendra, ICAR-Central Arid Zone Research Institute, Jodhpur, India; l Hasanand Gaushala, Vrindavan, Mathura, India; m Bovine Sperm Station and Research Centre, Hisar, India; n Department of Veterinary Pathology, Birsa Agricultural University, Ranchi, India; o Animal Science Division, Indian Council of Agricultural Research, India; p Centre for Animal Disease Research and Diagnosis, Indian Veterinary Research Institute, Izatnagar, India

**Keywords:** Lumpy skin disease, LSD/Ranchi, India, homologous live-attenuated LSD vaccine

## Abstract

Lumpy skin disease (LSD) was reported for the first time in India in 2019 and since then, it has become endemic. Since a homologous (LSD-virus based) vaccine was not available in the country, goatpox virus (GPV)-based heterologous vaccine was authorized for mass immunization to induce protection against LSD in cattle. This study describes the evaluation of safety, immunogenicity and efficacy of a new live-attenuated LSD vaccine developed by using an Indian field strain, isolated in 2019 from cattle. The virus was attenuated by continuous passage (*P* = 50) in Vero cells. The vaccine (50^th^ LSDV passage in Vero cells, named as Lumpi-ProVac^*Ind*^) did not induce any local or systemic reaction upon its experimental inoculation in calves (*n* = 10). At day 30 post-vaccination (pv), the vaccinated animals were shown to develop antibody- and cell-mediated immune responses and exhibited complete protection upon virulent LSDV challenge. A minimum Neethling response (0.018% animals; 5 out of 26,940 animals) of the vaccine was observed in the field trials conducted in 26,940 animals. There was no significant reduction in the milk yield in lactating animals (*n* = 10108), besides there was no abortion or any other reproductive disorder in the pregnant animals (*n* = 2889). Sero-conversion was observed in 85.18% animals in the field by day 30 pv.

## Introduction

Lumpy skin disease (LSD) is a transboundary animal disease which leads to heavy economic losses to the livestock industry. The disease is characterized by the development of skin nodules, fever, enlargement of lymph nodes, anorexia, depression, dysgalactia and emaciation which may eventually result in a sharp decline in the milk production, abortion in pregnant cows and infertility in bulls [1]. LSD is a serious hazard to the food security of the people in the affected areas [1,2]. The World Organization for Animal Health (WOAH) categorizes LSD as a notifiable disease. The disease has remained restricted to Africa ever since its first occurrence [3]. Its first intercontinental spread was confirmed in Israel in 1989 [4]. Since 2012, LSD has spread from Africa into several countries of Europe. It was first reported in the Asia and the Pacific region in 2019.

LSD was reported for the first time in India in 2019 [5]. Currently the country is facing the wrath of this deadly viral epidemic. The mortality rate in LSD is usually considered low (<1%) [6], however, it has been much higher during the current wave of LSD in India [1]. The loss of milk production in the affected cows has been reported to be between 26% and 50% [7].

LSD virus (LSDV) is a member of the genus *Capripoxvirus* within the family *Poxviridae*. LSDV genome is ~151 Kbp in length [8]. Sheeppox virus (SPV) and goatpox virus (GPV), two other capripoxviruses which cause severe disease in sheep and goats respectively, are also antigenically similar to LSDV [9]. Capripoxviruses are believed to provide cross protection within the genus; therefore SPV- or GPV-based vaccines are commonly used to induce cross protection against LSDV in cattle [9–13]. Based on this assumption (but without and experimental proof), the policy makers in India authorized the use of goatpox vaccine (heterologous vaccine) against LSD in cattle in 2021 [1,14]. However, the cross protection issue has been controversial [10,15,16]. There are numerous examples wherein GPV/SPPV-based vaccines have been shown to induce partial protection against LSDV in cattle [9,17–21]. These discrepancies in the use of heterologous vaccines in the past, together with the poor efficacy of goatpox vaccine in India, prompted us to develop a homologous vaccine which confers solid immunity against LSD in cattle [9,22]. This study describes evaluation of the safety, immunogenicity and efficacy of a new homologous live-attenuated LSD vaccine developed in India.

## Materials and methods

### Ethics statement

Vaccine efficacy experiments were conducted in calves at Indian Veterinary Research Institute (IVRI), Mukteswar, after obtaining due approval from the Committee for Purpose of Control and Supervision of Experiments on Animals (CPCSEA), Department of Animal Husbandry and Dairying, Ministry of Fisheries, Animal Husbandry and Dairying, Government of India (V-11011 (13)/3/2022/CPCSEA-DADF, dated 10.03.2022).

The field trials were approved by National Research Centre on Equines, Indian Council of Agricultural Research, Hisar, India. Due consent was taken from the concerned farmer for inoculation of the experimental vaccine in cattle/buffaloes.

### Cells

Primary lamb testicle cells [23] and African green monkey kidney (Vero) cells [24] were available at National Centre for Veterinary Type Cultures (NCVTC), Hisar and grown in Dulbecco’s Modified Eagle’s Medium (DMEM) supplemented with antibiotics and 10–15% foetal calf serum.

### Viruses

LSDV field strain used to develop live-attenuated LSD vaccine in this study was isolated by our group in 2019 from an outbreak in cattle at Ranchi (India) [5]. It was deposited in the national repository (NCVTC, Hisar, India) with Accession Number VTCCAVA 288 [5]. Hereinafter, it will be referred as LSDV/2019/RCH or LSDV/RCH/P0 or LSDV/P0 throughout the manuscript. Besides, cattle isolates from 2021 (Accession Number, VTCCAVA 321) and 2022 (Accession Number, VTCCAVA 370) and a camel isolate from 2022 (Accession Number, VTCCAVA 371) were also employed for cross neutralization studies.

### Attenuation of LSDV in Vero cells

In order to attenuate the virus (LSDV/2019/RCH) was sequentially passaged in Vero cells for up to 50 passages (P). Hereinafter, it will be interchangeably used as LSDV/RCH/P50 or LSDV/P50 or Indian vaccine strain. For each passage, 500 µl inoculum of the virus having a titre of ~10^6^ TCID_50_/ml from the previous passage was used to infect fresh Vero cells for 2 h, followed by washing with phosphate buffered saline (PBS) and addition of fresh growth medium. The virus was harvested when the cells exhibited ~75 % cytopathic effect (CPE).

### Whole genome sequencing

The P50 virus was completely sequenced at Next Generation Sequencing Platform (Clevergene Biocorp Pvt Ltd, Bengaluru, India). Viral DNA was extracted by DNeasy® Blood & Tissue Kit (Qiagen, Hilden, Germany) as per the instructions of the manufacturer and sent to Clevergene Biocorp Pvt Ltd (Bengaluru, India) for whole-genome sequencing. The complete nucleotide sequences of wild type (LSDV/P0) and LSDV/P50 viruses were deposited to GenBank Accession with Number MW883897.1 and OK422494.1 respectively and the mutations were identified by using an online tool (https://www.ebi.ac.uk/Tools/msa/clustalo/).

### Virus neutralization assay

Serum samples were heated at 56°C for 30 min to inactivate the complements. Vero cells were grown in 96 well tissue culture plates till ~90 confluency. Two-fold serum dilutions (in 50 µl volume) were made in PBS and incubated with an equal volume of ~10 [2] TCID_50_ of LSDV for 1 h at 37°C. The virus-antibody mixture was then used to infect Vero cells. The cells were daily observed under the microscope for the appearance of CPE. Final reading was taken at 72 hours post-infection (hpi) for the determination of antibody titres.

### Vaccine preparation

The final vaccine preparation was carried out by diluting the original virus stock of LSDV/P50 (~10^7^ TCID_50_/ml) in sterile PBS to prepare aliquots of 10^4.5^ TCID_50_/ml (10× field dose) and 10^3.5^ TCID_50_/ml (1× field dose). The virus (vaccine) was tested for its sterility, wherein two ml of the final vaccine preparation was inoculated in 10 ml of Fluid thioglycollate medium (FTM) at 30°–35°C (for anaerobes) and Tryptic soy broth (TSB) at 20°–25°C (for aerobe/fungi) for 10 d. The sterility was ascertained by the absence of microbial contamination for up to 14 d. Besides, the final vaccine preparation was also tested for *Mycoplasma sps* and bovine viral diarrhoea virus (BVDV) by amplification of their respective gene segments in PCR.

### qRT-PCR

DNA was isolated from blood and nasal/fecal/ocular swab by DNeasy Blood & Tissue Kit (Qiagen, Hilden, Germany). The viral DNA was detected by TaqMan-probe-based real-time quantitative PCR as per the previously described method [25]. The primers flanked a conserved 151-bp region of LSDV044 target region (forward primer-5’- CAA AAACAATCGTAACTAATCCA −3;’ reverse primer-5’- TGGAGTTTTTATGTCATCGTC-3’). The probe (5’-6-FAM-TCGTCGTCGTTTAAAACTGA- QSY-3’) was labelled with 6-carboxy fluorescein (FAM), the reporter dye at the 5′-end and the QSY quencher at the 3′-end. Each 20-μL PCR reaction comprised 5 μL of DNA, 10 μL of 2 × Taq Man Universal PCR Master mix (Thermo Fisher Scientific, USA), 10 μM of each primer and 2.5 μM of the probe. PCR was conducted on a QuantStudio 3 thermal cycler (Thermo Fisher Scientific, USA) with following Thermalcycler conditions – 95°C for 5 min, followed by 40 cycles of 95°C for 15 s, and 60°C for 60 s. Threshold cycle (Ct) values of ≤35 were considered as positive.

### Differentiation of LSDV vaccine and field (virulent) strains

LSDV ORF44 (*zdf4ln*) contains a conserved region which is present in all the LSDV strains but not in other capripoxviruses. This was exploited to specifically amplify LSDV genome (not SPV and GPV) by TaqMan real-time PCR as per the previously described method [25].

As compared to the field/virulent LSDV strains, most LSDV vaccine strains including Indian vaccine strain (developed in this study) contain a 12 bp insertion in its GPCR gene. This was exploited to specifically amplify the vaccine strain (but not field strains) by TaqMan real-time PCR as per the previously described method [26].

### Preparation of challenge virus

Skin nodules were collected from a naturally LSDV-infected cattle. A 10% suspension of skin nodules was used to infect the primary goat kidney cells. At 4 d following infection, the cells were freeze-thawed twice to harvest the virus (P1). The P1 virus was bulk cultured in primary lamb testicle cells and concentrated 50-times by ultracentrifugation. The final virus preparation (P2) had a virus titre of 10^5.5^ TCID_50_/ml (Ct value of 19.6 in qRT-PCR).

### Safety and efficacy

The safety and efficacy was conducted as per WOAH Terrestrial Manual 2021 [27]. LSDV seronegative male calves, aged 6–9 months, were included in the study. Out of the 15 calves used to evaluate the safety and efficacy of the vaccine, two animals were inoculated with 10^4.5^ TCID_50_ while eight were inoculated with 10^3.5^ TCID_50_ and 5 were kept as unvaccinated control.

Animals were monitored for any clinical signs and rectal temperatures were recorded. At day 30 pv, the animals were challenged with virulent virus by intraveneous (2 ml) and intradermal (0.25 ml at four sites on the flank region) routes. The clinical response was recorded for 17 d.

### Safety in the field animals

A total of 26,940 animals (cattle and buffaloes) of all age groups including lactating (*n* = 10108) and pregnant (2889) animals were vaccinated with a recommended field dose (10^3.5^ TCID_50_) of the vaccine by subcutaneous route. All the animals were observed for development of any local or systemic reactions. Body temperature was recorded in selected farms. Total daily milk production in lactating animals and reproductive disorders (abortion) in pregnant animals were also recorded.

### Cell proliferation assay

Peripheral blood mononuclear cells (PBMCs) were isolated from blood by HISTOPAQUE®-1077 (Sigma, Steinheim, Germany) as per the instructions of the manufacturer and resuspended in RPMI (Sigma, St Luis, USA) supplemented with 15% FBS. The cells were cultured in 96-well tissue culture plates at concentrations of 2 × 10^6^ cells/ml in 100 μl volume and stimulated with either 10 µg of UV-inactivated LSDV antigen- or Concavalin A (positive control). A negative control was made up of unstimulated PBMCs in cell culture medium only. Cultures were incubated at 37°C in 5% CO_2_ for 72 h. Five milligrams of MTT was dissolved into 1 ml of PBS, with 50 μl added onto each well containing cells, and incubated at 37°C for 4 h. Finally 100 µl of DMSO was added to dissolve the formazan crystals. The absorbance (OD) was measured at 570 nm and relative increase in cell numbers in LSDV-antigen stimulated over un-stimulated wells was determined.

### Measurement of IFN-ϒ

The levels of IFN-ϒ in naïve, vaccinated, vaccinated-challenged and unvaccinated-challenged animals were measured by Bovine IFN-ϒ-ELISA kit (Invitrogen, Frederick, USA) as per the instructions of the manufacturer.

### Statistical analysis

Effect of vaccination on different biological parameters in vaccinated and control animals was compared using Student’s t-test

## Results

### Genetic signatures of vaccine virus (LSDV/P50)

Like other poxviruses, *Capripoxvirus* genome is quite large (151 Kbp) and undergoes extensive mutations during cell culture passage. Besides several point- and frame-shift mutations, insertions and deletions of DNA segments can also be observed during the evolution of *Capripoxviruses* [28,29]. We compared the whole genome sequences of LSDV/2019/RCH at P0 (field strain, GenBank Accession Number MW883897) and at P50 levels (50^th^ Vero passage, GenBank Accession Number OK422494). As compared to the LSDV/P0, the major mutations in LSDV/P50 were observed in genes encoding Ankyrin repeat proteins, Kelch-like proteins, EEV membrane phosphoglycoprotein and DNA-dependent RNA polymerase. Nevertheless, these viral genes have also been shown to be disrupted in other live-attenuated vaccine candidates of capripoxviruses (LSDV, SPV and GPV) [29]. A twelve bps insertion in G-protein coupled chemokine receptor (GPCR)-the signature mutation of most of the *Capripoxvirus* vaccine strains [26] was also present in LSDV/P50. Interestingly, in contrast to other existing field and vaccine strains, a unique deletion of 801 bp in the inverted terminal repeat region (ITR) was also observed in LSDV/RCH/P50.

### Preparation of vaccine

**Figure 1.**
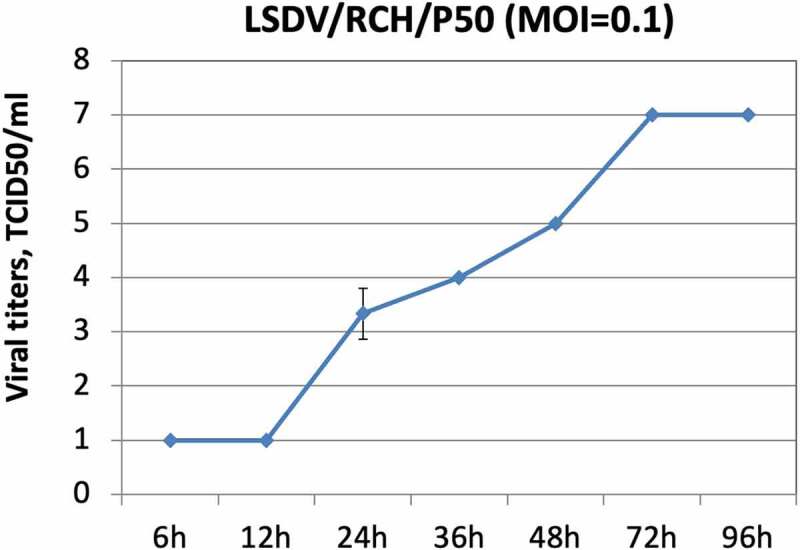
**Growth characteristics of LSDV/RCH/P50**. Vero cells, in triplicates, were infected with LSDV/RCH/P50 at MOI of 0.1 for 2 h, followed by washing with PBS and addition of fresh DMEM. Supernatant was collected from the infected cells at indicated time points and quantified by determination of TCID_50_ in Vero cells. Values are means ± SD and representative of the result of at least 3 independent experiments.

The LSDV/P50 produced CPE within 2–3 d (depending on MOI used to infect). The optimum yield (10^7^ TCID_50_/ml) of LSDV/P50 in Vero cells was obtained at MOI of 0.1 and at a virus harvest time of 72 h (Figure 1). Besides sterility, the virus stock was also tested negative for extraneous agents viz; *Mycoplasma sps* and Bovine viral diarrhoea virus. The experimental trials were conducted with frozen stock of LSDV/P50. For the field trials, freeze-dried virus (vaccine) was used. To prepare freeze dried stock (50 doses pack size), the original stock of LSDV/P50 (10^7^ TCID_50_/ml) was diluted 100-times in PBS and 1 ml of it was dispensed into each vial (10^5.5^ TCID_50_/ml per 50 doses). Since freeze-drying resulted in a loss of~5-fold virus titre, 5-times higher concentration (10^5.5^ TCID_50_/ml per 50 doses; 10^4^ TCID_50_/ml per dose) was considered for freeze-drying. The freeze-dried vaccine had a virus titre of 10^3.5^ TCID_50_/dose. It was sterile and free from extraneous agents and named as Lumpi-ProVac^Ind^.

### Safety (Experimental trial)

Vaccinated calves did not develop any local or systemic reaction. Rise in temperature was recorded in a total of three animals viz; day 1 post-vaccination (pv) (IVRI 1529), day 3 pv (IVRI 1541 and IVRI 1529) day 12 pv (IVRI 1519) and day 15 pv (IVRI 1529) **(Supplementary Table S1)**. Viral genome was detected in the blood in some of the vaccinated animals at day 3 pv (Table 1), however, the virus could not be isolated in any of the animals. Nasal, ocular and faecal shedding was not reported in any of the immunized animals up to day 30 pv (Table 1). All the immunized animals remained apparently healthy with a normal feed intake without exhibiting any untoward reaction. Various haematological and blood biochemical parameters of vaccinated (*n* = 10) and unvaccinated (*n* = 5) animals were also comparable **(Supplementary Figure S1 and S2)**.

**Table 1.** Observation of various safety parameters following vaccination with Lumpi-ProVac^Ind^ (Experimental trial).

		Days post-vaccination								
Animal	Dose		0	3	6	9	12	15	18	30
IVRI-1519	10X	Antibody titre	<4	NT	NT	<4	NT	NT	<4	16
		IFN-ϒ	-Ve	+Ve	-Ve	NT	NT	NT	NT	NT
		Skin nodules	-Ve	-Ve	-Ve	-Ve	-Ve	-Ve	-Ve	-Ve
		Viraemia	-Ve	-Ve	-Ve	-Ve	NA	NA	NA	NA
		G-Nasal	-Ve	-Ve	-Ve	-Ve	-Ve	-Ve	-Ve	-Ve
		G-Ocular	-Ve	-Ve	-Ve	-Ve	-Ve	-Ve	-Ve	-Ve
		G-Fecal	-Ve	-Ve	-Ve	-Ve	-Ve	-Ve	-Ve	-Ve
IVRI-1552	10X	Antibody titre	<4	NT	NT	<4	NT	NT	<4	16
		IFN-ϒ	-Ve	+Ve	-Ve	NT	NT	NT	NT	NT
		Skin nodules	-Ve	-Ve	-Ve	-Ve	-Ve	-Ve	-Ve	-Ve
		Viraemia	-Ve	-Ve*	-Ve	-Ve	NT	NT	NT	NT
		G-Nasal	-Ve	-Ve	-Ve	-Ve	-Ve	-Ve	-Ve	-Ve
		G-Ocular	-Ve	-Ve	-Ve	-Ve	-Ve	-Ve	-Ve	-Ve
		G-Fecal	-Ve	-Ve	-Ve	-Ve	-Ve	-Ve	-Ve	-Ve
IVRI-1550	1X	Antibody titre	<4	NA	NA	<4	NA	NA	8	8
		IFN-ϒ	-Ve	-Ve	-Ve	NT	NT	NT	NT	NT
		Skin nodules	-Ve	-Ve	-Ve	-Ve	-Ve	-Ve	-Ve	-Ve
		Viraemia	-Ve	-Ve	-Ve	-Ve	NT	NT	NT	NT
		G-Nasal	-Ve	-Ve	-Ve	-Ve	-Ve	-Ve	-Ve	-Ve
		G-Ocular	-Ve	-Ve	-Ve	-Ve	-Ve	-Ve	-Ve	-Ve
		G-Fecal	-Ve	-Ve	-Ve	-Ve	-Ve	-Ve	-Ve	-Ve
IVRI-1541	1X	Antibody titre	<4	NT	NT	<4	NT	NT	<4	<4
		IFN-ϒ	-Ve	-Ve	-Ve	NT	NT	NT	NT	NT
		Skin nodules	-Ve	-Ve	-Ve	-Ve	-Ve	-Ve	-Ve	-Ve
		Viraemia	-Ve	-Ve	-Ve	-Ve	NT	NT	NT	NT
		G-Nasal	-Ve	-Ve	-Ve	-Ve	-Ve	-Ve	-Ve	-Ve
		G-Ocular	-Ve	-Ve	-Ve	-Ve	-Ve	-Ve	-Ve	-Ve
		G-Fecal	-Ve	-Ve	-Ve	-Ve	-Ve	-Ve	-Ve	-Ve
IVRI-1533	1X	Antibody titre	<4	NT	NT	<4	NT	NT	<4	32
		IFN-ϒ	-Ve	-Ve	-Ve	NT	NT	NT	NT	NT
		Skin nodules	-Ve	-Ve	-Ve	-Ve	-Ve	-Ve	-Ve	-Ve
		Viraemia	-Ve	-Ve*	-Ve	-Ve	NT	NT	NT	NT
		G-Nasal	-Ve	-Ve	-Ve	-Ve	-Ve	-Ve	-Ve	-Ve
		G-Ocular	-Ve	-Ve	-Ve	-Ve	-Ve	-Ve	-Ve	-Ve
		G-Fecal	-Ve	-Ve	-Ve	-Ve	-Ve	-Ve	-Ve	-Ve
IVRI-1462	1X	Antibody titre	8	NT	NT	16	NT	NT	16	32
		IFN-ϒ	+Ve	+Ve	-Ve	NT	NT	NT	NT	NT
		Skin nodules	-Ve	-Ve	-Ve	-Ve	-Ve	-Ve	-Ve	-Ve
		Viraemia	-Ve	-Ve*	-Ve	-Ve	NA	NA	NA	NA
		G-Nasal	-Ve	-Ve	-Ve	-Ve	-Ve	-Ve	-Ve	-Ve
		G-Ocular	-Ve	-Ve	-Ve	-Ve	-Ve	-Ve	-Ve	-Ve
		G-Fecal	-Ve	-Ve	-Ve	-Ve	-Ve	-Ve	-Ve	-Ve
IVRI-1489	1X	Antibody titre	<4	NT	NT	<4	NT	NT	<4	64
		IFN-ϒ	-Ve	-Ve	-Ve	NT	NT	NT	NT	NT
		Skin nodules	-Ve	-Ve	-Ve	-Ve	-Ve	-Ve	-Ve	-Ve
		Viraemia	-Ve	-Ve*	-Ve	-Ve	NT	NT	NT	NT
		G-Nasal	-Ve	-Ve	-Ve	-Ve	-Ve	-Ve	-Ve	-Ve
		G-Ocular	-Ve	-Ve	-Ve	-Ve	-Ve	-Ve	-Ve	-Ve
		G-Fecal	-Ve	-Ve	-Ve	-Ve	-Ve	-Ve	-Ve	-Ve
IVRI-1524	1X	Antibody titre	<4	NT	NT	<4	NT	NT	<4	32
		IFN-ϒ	-Ve	+Ve	+Ve	+Ve	NT	NT	NT	NT
		Skin nodules	-Ve	-Ve	-Ve	-Ve	-Ve	-Ve	-Ve	-Ve
		Viraemia	-Ve	-Ve*	-Ve	-Ve	NT	NT	NT	NT
		G-Nasal	-Ve	-Ve	-Ve	-Ve	-Ve	-Ve	-Ve	-Ve
		G-Ocular	-Ve	-Ve	-Ve	-Ve	-Ve	-Ve	-Ve	-Ve
		G-Fecal	-Ve	-Ve	-Ve	-Ve	-Ve	-Ve	-Ve	-Ve
IVRI-1529	1X	Antibody titre	<4	NT	NT	<4	NT	NT	8	16
		IFN-ϒ	-Ve	+Ve	-Ve	NT	NT	NT	NT	NT
		Skin nodules	-Ve	-Ve	-Ve	-Ve	-Ve	-Ve	-Ve	-Ve
		Viraemia	-Ve	-Ve	-Ve	-Ve	NT	NT	NT	NT
		G-Nasal	-Ve	-Ve	-Ve	-Ve	-Ve	-Ve	-Ve	-Ve
		G-Ocular	-Ve	-Ve	-Ve	-Ve	-Ve	-Ve	-Ve	-Ve
		G-Fecal	-Ve	-Ve	-Ve	-Ve	-Ve	-Ve	-Ve	-Ve
IVRI-745	1X	Antibody titre	<4	NT	NT	<4	NT	NT	<4	16
		IFN-ϒ	-Ve	-Ve	-Ve	NT	NT	NT	NT	NT
		Skin nodules	-Ve	-Ve	-Ve	-Ve	-Ve	-Ve	-Ve	-Ve
		Viraemia	-Ve	-Ve*	-Ve	-Ve	NT	NT	NT	NT
		G-Nasal	-Ve	-Ve	-Ve	-Ve	-Ve	-Ve	-Ve	-Ve
		G-Ocular	-Ve	-Ve	-Ve	-Ve	-Ve	-Ve	-Ve	-Ve
		G-Fecal	-Ve	-Ve	-Ve	-Ve	-Ve	-Ve	-Ve	-Ve

(−Ve): Negative; (NT): Not tested; (*):Positive in viral genome but virus could not be isolated; (G-Nasal): Viral genome in nasal secretion; (G-Ocular): Viral genome in ocular secretion; (G-Faecal): Viral genome in faecal secretion.

### Safety in the field animals

**Figure 2.**
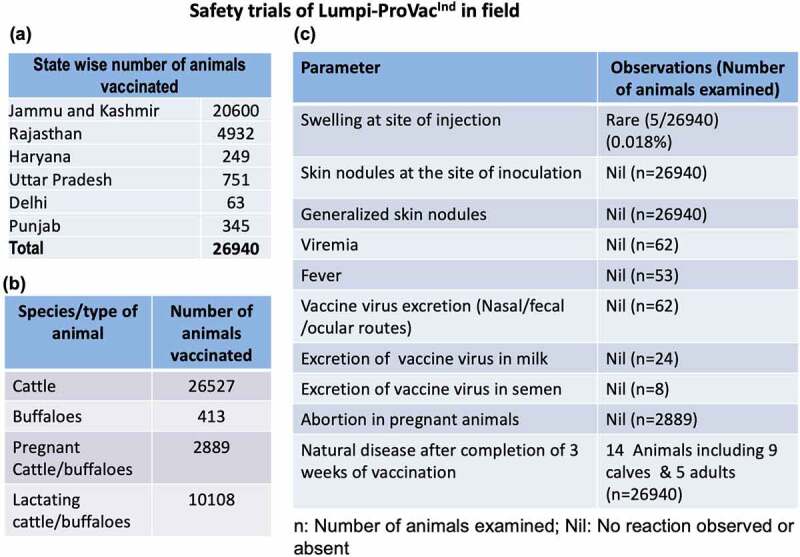
**Safety of Lumpi-ProVacInd in field animals**. a total of 26,940 animals across six Indian states **(a)** comprising of 26,527 cattle, 413 buffaloes (2889 pregnant cattle/buffaloes and 10,108 lactating buffaloes) **(b)** were included in the study. All the animals were injected with 1 ml of Lumpi-ProVac^Ind^ (containing 10^3.5^ TCID_50_/dose) by subcutaneous route and monitored for swelling/skin nodules at the site of injection, generalized skin nodules, abortions in pregnant animals and efficacy of the vaccine **(c)**.

A total of 26,940 animals (26527 cattle and 413 buffaloes) across six different states in India (Figure 2a,b) were included in the study. A single field dose of the vaccine (10^3.5^ TCID_50_) was found to be safe in cattle and buffaloes of all age groups including calves, pregnant/lactating animals and bulls. Fever was not observed in the field animals, although daily body temperature was noted in selected farms **(Supplementary Table S2 and S3)**. Very mild swelling response (local site reaction which appeared at day 3 pv and subsided within 2 d without any specific intervention/treatment) was observed in 5 out of the 26,940 vaccinated animals (Table 2, Figure 2c). Generalized skin nodules were not observed in any of the vaccinated animals. Likewise, abortion or any other reproductive disorders were also not observed in any of the 2889 pregnant (3 to 9 months of pregnancy) animals that received the vaccine (Table 2). Out of the 102 farms/units included in the study, a slight (non-significant) drop in milk production was recorded only in 4 farms/units (235 litres out of a total of 536,024 litres in 8 d) (Table 3). However, this drop in milk production was temporary and was regained within 2–8 d pv (Table 3). All the vaccinated animals remained apparently healthy following vaccination without any significant alteration in feed/water intake.

**Table 2.** Safety and efficacy of Lumpi-ProVac^Ind^ in field animals.

S. N.	Farm/Unit	Number of animals vaccinated	Local reaction	Skin nodules	Number of Pregnant Animals$	Number of abortions	Disease (3 weeks after vaccination)	Mortality (3 weeks after vaccination)
1	LPV FT-J&K F1	600	0	0	30	0	0	0
2	LPV FT-J&K F2	100	0	0	0	0	0	0
3	LPV FT-J&K F3	700	0	0	146	0	0	0
4	LPV FT-J&K F4	200	0	0	22	0	0	0
5	LPV FT-J&K F5	200	0	0	26	0	0	0
6	LPV FT-J&K F6	100	0	0	12	0	0	0
7	LPV FT-J&K F7	100	0	0	8	0	0	0
8	LPV FT-J&K F8	400	0	0	37	0	0	0
9	LPV FT-J&K F9	500	0	0	27	0	0	0
10	LPV FT-J&K F10	200	0	0	21	0	0	0
11	LPV FT-J&K F11	500	0	0	30	0	0	0
12	LPV FT-J&K F12	100	0	0	26	0	0	0
13	LPV FT-J&K F13	300	0	0	60	0	0	0
14	LPV FT-J&K F14	200	0	0	40	0	0	0
15	LPV FT-J&K F15	500	0	0	40	0	0	0
16	LPV FT-J&K F16	500	0	0	35	0	0	0
17	LPV FT-J&K F17	500	0	0	4	0	0	0
18	LPV FT-J&K F18	2000	0	0	203	0	0	0
19	LPV FT-J&K F19	1000	0	0	76	0	0	0
20	LPV FT-J&K F20	300	0	0	0	0	0	0
21	LPV FT-J&K F21	500	0	0	27	0	0	0
22	LPV FT-J&K F22	100	0	0	3	0	0	0
23	LPV FT-J&K F23	300	0	0	20	0	0	0
24	LPV FT-J&K F24	300	0	0	15	0	0	0
25	LPV FT-J&K F25	200	0	0	0	0	0	0
26	LPV FT-J&K F26	700	0	0	17	0	0	0
27	LPV FT-J&K F27	600	0	0	50	0	0	0
28	LPV FT-J&K F28	400	0	0	22	0	0	0
29	LPV FT-J&K F29	600	0	0	79	0	0	0
30	LPV FT-J&K F30	200	0	0	0	0	0	0
31	LPV FT-J&K F31	200	0	0	10	0	0	0
32	LPV FT-J&K F32	200	0	0	28	0	0	0
33	LPV FT-J&K F33	100	0	0	10	0	0	0
34	LPV FT-J&K F34	200	0	0	30	0	0	0
35	LPV FT-J&K F35	100	0	0	12	0	0	0
36	LPV FT-J&K F36	200	0	0	0	0	0	0
37	LPV FT-J&K F37	200	0	0	35	0	0	0
38	LPV FT-J&K F38	200	0	0	0	0	0	0
39	LPV FT-J&K F39	200	0	0	0	0	0	0
40	LPV FT-J&K F40	200	0	0	30	0	0	0
41	LPV FT-J&K F41	200	0	0	20	0	0	0
42	LPV FT-J&K F42	100	0	0	19	0	0	0
43	LPV FT-J&K F43	100	0	0	21	0	0	0
44	LPV FT-J&K F44	200	0	0	13	0	0	0
45	LPV FT-J&K F45	100	0	0	26	0	0	0
46	LPV FT-J&K F46	200	0	0	30	0	0	0
47	LPV FT-J&K F47	200	0	0	30	0	0	0
48	LPV FT-J&K F48	200	0	0	55	0	0	0
49	LPV FT-J&K F49	300	0	0	60	0	0	0
50	LPV FT-J&K F50	200	0	0	41	0	0	0
51	LPV FT-J&K F51	100	0	0	19	0	0	0
52	LPV FT-J&K F52	150	0	0	32	0	0	0
53	LPV FT-J&K F53	300	0	0	65	0	0	0
54	LPV FT-J&K F54	200	0	0	40	0	0	0
55	LPV FT-J&K F55	100	0	0	15	0	0	0
56	LPV FT-J&K F56	300	0	0	20	0	0	0
57	LPV FT-J&K F57	450	0	0	47	0	0	0
58	LPV FT-J&K F58	200	0	0	18	0	0	0
59	LPV FT-J&K F59	200	0	0	0	0	0	0
60	LPV FT-J&K F60	200	0	0	20	0	0	0
61	LPV FT-J&K F61	200	0	0	30	0	0	0
62	LPV FT-J&K F62	200	0	0	NA	0	0	0
63	LPV FT-J&K F63	400	0	0	112	0	0	0
64	LPV FT-J&K F64	400	0	0	65	0	0	0
65	LPV FT-J&K F65	200	0	0	25	0	0	0
66	LPV FT-J&K F66	300	0	0	47	0	0	0
67	LPV FT-J&K F67	200	0	0	27	0	0	0
68	LPV FT Raj-1	140	0	0	12	0	0	0
69	LPV FT Raj-2	35	1	0	17	0	0	0
70	LPV FT Raj-3	184	0	0	5	0	0	0
71	LPV FT Raj-4	148	0	0	38	0	0	0
72	LPV FT Raj-5	163	0	0	19	0	0	0
73	LPV FT Raj-6	114	2	0	22	0	1 (Calf)	0
74	LPV FT Raj-7	498	0	0	15	0	5 Adults & 1 Calf	0
75	LPV FT Raj-8	150	0	0	18	0		0
76	LPV FT Raj-9	62	0	0	16	0	0	0
77	LPV FT Raj-10	582	0	0	38	0	0	0
78	LPV FT Raj-11	424	0	0	16	0	0	0
79	LPV FT Raj-12	310	0	0	18	0	0	0
80	LPV FT Raj-13	723	0	0	37	0	0	0
81	LPV FT Raj-14	42	0	0	2	0	0	0
82	LPV FT Raj-15	50	0	0	5	0	0	0
83	LPV FT Raj-16	11	0	0	4	0	0	0
84	LPV FT Raj-17	164	0	0	126	0	0	0
85	LPV FT Raj-18	10	0	0	8	0	1 (Calf)	0
86	LPV FT Raj-19	18	0	0	4	0	0	0
87	LPV FT Raj-20	127	0	0	7	0	0	0
88	LPV FT Raj-21	97	0	0	31	0	0	0
89	LPV FT Raj-22	150	0	0	42	0	0	0
90	LPV FT Raj-23	115	0	0	19	0	0	0
91	LPV FT Raj-24	200	0	0	22	0	0	0
92	LPV FT Raj-25	189	0	0	11	0	0	0
93	LPV FT Raj-26	150	0	0	45	0	0	0
94	LPV FT Raj-27	37	0	0	5	0	0	0
95	LPV FT Raj-28	39	0	0	10	0	0	0
96	LPV FT Hry 1	100	0	0	0	0	0	0
97	LPV FT Hry 2	125	2	0	15	0	1 (Calf)	0
98	LPV FT Hry 3	7	0	0	2	0	0	0
99	LPV FT Hry 4	17	0	0	7	0	0	0
100	LPV FT UP 1	751	0	0	94	0	5 (Calves)	0
101	LPV FT Del 1	63	0	0	31	0	0	0
102	LPV FT Pub 1	345	0	0	99	0	0	0
	Total number of animals	26940	5	0	2889	0	14	0

$: Pregnant animals were 3–9 months of pregnancy

**Table 3.** Effect of Lumpi-ProVac^Ind^ on milk production.

S. N.	Farm/Unit	Number of lactating animals	Total milk production/day (ltr)	Total milk production in 8 days (ltr)	Total milk reduction observed in 8 days (ltr)	Regain of milk yield (d)
1	LPV FT Raj-6	32	250	2000	45	2 d
2	LPV FT Raj-24	8	40	320	40	4 d
3	LPV FT Raj-26	32	180	1440	45	5 d
4	LPV FT Hry 2	75	150	1200	105	8 d
(Affected/Total)	4 (102)	147 (10108)	620 (67003)	4960 (536024)	235#	-

#=Non-significant reduction in milk production. Out of the 102 farms registered, reduced milk production was observed only in 4 farms for a maximum period of 8 d. A reduction of 235 ltr milk was observed in 4 affected farms in 8 d which was non-significant (102 farms with a total production of 536,024 ltr in 8 d).

### Immunogenicity

**Figure 3.**
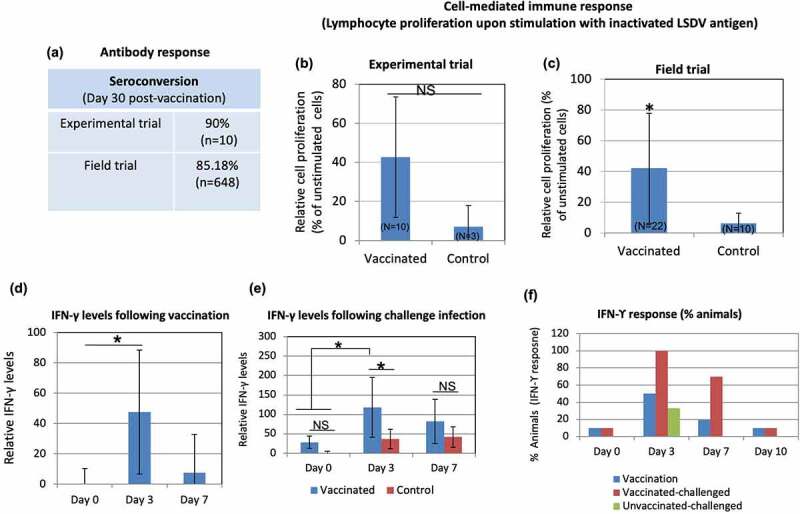
**Immunogenicity of Lumpi-ProVacInd**. Immunogenicity was evaluated in experimental animals (*n*=10) and selected field animals (*n*=22). **(a) Antibody titers**. Percentage of animals (experimental and field trials) that revealed detectable anti-LSDV antibodies in serum by virus neutralization assay is shown. **(b) Lymphocyte proliferation assay (experimental trial)**. PBMCs were separated from the blood collected from vaccinated (*n*=10) or unvaccinated (*n*=3) calves at day 30 pv. PBMCs were cultured in RPMI and stimulated with UV-inactivated LSDV. Relative proliferation of lymphocyte from vaccinated as compared to unvaccinated animals is shown. **(c) Lymphocyte proliferation assay (field trial)**. PBMCs were separated from the blood collected from vaccinated (*n*=22) or unvaccinated (*n*=10) animals (all age groups) at day 30 pv and stimulated with UV-inactivated LSDV. Relative proliferation of lymphocyte from vaccinated as compared to the unvaccinated animals is shown. **(d) IFN-ϒ levels following vaccination**. Sera from experimental calves, separated at indicated times post-vaccination were subjected for determination of IFN-ϒ by using Bovine IFN-ϒ-ELISA kit. **(e) IFN-ϒ levels following challenge infection**. Sera from vaccinated, vaccinated-challenged and unvaccinated-challenged were examined for determination of IFN-ϒ. **(f) Percentage of animals that developed IFN-ϒ response**. Percentage of animals that exhibited IFN-ϒ response at indicated times following LSDV exposureis shown. *=p<0.05, NS=non-significant difference.

Serology of vaccinated animals, evaluated by virus neutralization test (VNT) revealed 90% positive animals at day 30 pv (experimental trial) (Table 1, Figure 3a). Antibodies were observed starting from day 18 pv. At 30 day pv, the antibody titer ranged between 8 and 64 (Table 1). One of the cattle (IVRI 1541) did not reveal detectable amount of antibodies up to day 30 pv (Table 1). Although, prior to the start of experiment, animals were screened for seronegativity (free from antibodies against capripoxviruses) but one animal (IVRI 1462) had pre-existing antibodies at day 0 pv. In field trials, seroconversion was detected in 85.18% animals (n = 648) (Figure 3a).

We also evaluated the cell-mediated immune response by lymphocyte proliferation assay and detection of IFN-ϒ in serum. At day 30 pv, as compared to the unvaccinated controls, PBMCs from 60% of the immunized animals showed a significant cell proliferation response following stimulation with UV-inactivated LSDV antigen (Figure 3), although due to large animal-to-animal variation, the overall relative difference in cell proliferation between vaccinated and unvaccinated groups was non-significant (Figure 3b). However, in field trials, a significant difference in cell proliferative response was observed between vaccinated (*n* = 22) and unvaccinated (*n* = 10) animals (Figure 3c).

The IFN-ϒ levels were significantly higher in vaccinated as compared to the unvaccinated animals at day 3 pv (Figure 3d). Likewise, higher levels of IFN-ϒ were also observed in vaccinated-challenged as compared to unvaccinated-challenged or control (naïve) animals (Figure 3e). Following vaccination, the IFN-ϒ response was observed in 10%, 50%, 10% and 0% animals on day 0, day 3 day 7 and day 10 pv respectively (Table 4, Figure 3f). The challenge of vaccinated animals (*n* = 10) showed IFN-ϒ response in 0%, 100%, 70% and 10% animals on day 0, day 3, day 7 and day 10 post-challenge (pc) respectively (Table 4, Figure 3f). Among the unvaccinated-challenged group (*n* = 3), the IFN-ϒ response was observed in 0%, 66%, 33% and 0% animals on day 0, day 3, day 7 and day 10 pc respectively (Table 4, Figure 3f). To summarise, the highest IFN-ϒ response was observed at day 3 following exposure with LSDV, irrespective of vaccination or challenge.

**Table 4.** Efficacy of Lumpi-ProVac^Ind^ in cattle (Experimental challenge).

			Days post-challenge														
Animal$	Dose		0	1	2	3	4	5	6	7	8	9	10	11	12	13	14
IVRI-1519	10X	Antibody titer	16	NT	NT	NT	NT	NT	NT	NT	NT	NT	NT	NT	NT	NT	64
		Lymphoproliferation	***	NT	NT	NT	NT	NT	NT	NT	NT	NT	NT	NT	NT	NT	NT
		IFN-ϒ	-Ve	NT	NT	+Ve	NT	NT	NT	+Ve	NT	NT	-Ve	NT	NT	NT	NT
		Viraemia	-Ve	-Ve	-Ve	-Ve	-Ve	-Ve	-Ve	-Ve	-Ve	-Ve	-Ve	-Ve	-Ve	-Ve	-Ve
		Skin nodules	-Ve	-Ve	-Ve	-Ve	-Ve	-Ve	-Ve	-Ve	-Ve	-Ve	-Ve	-Ve	-Ve	-Ve	-Ve
IVRI-1552	10X	Antibody titer	16	NT	NT	NT	NT	NT	NT	NT	NT	NT	NT	NT	NT	NT	64
		Lymphoproliferation	**	NT	NT	NT	NT	NT	NT	NT	NT	NT	NT	NT	NT	NT	NT
		IFN-ϒ	-Ve	NT	NT	+Ve	NT	NT	NT	+Ve	NT	NT	-Ve	NT	NT	NT	NT
		Viraemia	-Ve	-Ve	-Ve	-Ve	-Ve	-Ve	-Ve	-Ve	-Ve	-Ve	-Ve	-Ve	-Ve	-Ve	-Ve
		Skin nodules	-Ve	-Ve	-Ve	-Ve	-Ve	-Ve	-Ve	-Ve	-Ve	-Ve	-Ve	-Ve	-Ve	-Ve	-Ve
IVRI-1550	1X	Antibody titer	8	NT	NT	NT	NT	NT	NT	NT	NT	NT	NT	NT	NT	NT	32
		Lymphoproliferation	*	NT	NT	NT	NT	NT	NT	NT	NT	NT	NT	NT	NT	NT	NT
		IFN-ϒ	-Ve	NT	NT	+Ve	NT	NT	NT	+Ve	NT	NT	-Ve	NT	NT	NT	NT
		Viraemia	-Ve	-Ve	-Ve	-Ve	-Ve	-Ve	-Ve	-Ve	-Ve	-Ve	-Ve	-Ve	-Ve	-Ve	-Ve
		Skin nodules	-Ve	-Ve	-Ve	-Ve	-Ve	-Ve	-Ve	-Ve	-Ve	-Ve	-Ve	-Ve	-Ve	-Ve	-Ve
IVRI-1541	1X	Antibody titer	<4	NT	NT	NT	NT	NT	NT	NT	NT	NT	NT	NT	NT	NT	64
		Lymphoproliferation	*	NT	NT	NT	NT	NT	NT	NT	NT	NT	NT	NT	NT	NT	NT
		IFN-ϒ	-Ve	NT	NT	+Ve	NT	NT	NT	+Ve	NT	NT	-Ve	NT	NT	NT	NT
		Viraemia	-Ve	-Ve	-Ve	-Ve	-Ve	-Ve	-Ve	-Ve	-Ve	-Ve	-Ve	-Ve	-Ve	-Ve	-Ve
		Skin nodules	-Ve	-Ve	-Ve	-Ve	-Ve	-Ve	-Ve	-Ve	-Ve	-Ve	-Ve	-Ve	-Ve	-Ve	-Ve
IVRI-1533	1X	Antibody titer	32	NT	NT	NT	NT	NT	NT	NT	NT	NT	NT	NT	NT	NT	128
		Lymphoproliferation	**	NT	NT	NT	NT	NT	NT	NT	NT	NT	NT	NT	NT	NT	NT
		IFN-ϒ	-Ve	NT	NT	+Ve	NT	NT	NT	+Ve	NT	NT	-Ve	NT	NT	NT	NT
		Viraemia	-Ve	-Ve	-Ve	-Ve	-Ve	-Ve	-Ve	-Ve	-Ve	-Ve	-Ve	-Ve	-Ve	-Ve	-Ve
		Skin nodules	-Ve	-Ve	-Ve	-Ve	-Ve	-Ve	-Ve	-Ve	-Ve	-Ve	-Ve	-Ve	-Ve	-Ve	-Ve
IVRI-1462	1X	Antibody titer	32	NT	NT	NT	NT	NT	NT	NT	NT	NT	NT	NT	NT	NT	256
		Lymphoproliferation	NS	NT	NT	NT	NT	NT	NT	NT	NT	NT	NT	NT	NT	NT	NT
		IFN-ϒ	+Ve	NT	NT	+Ve	NT	NT	NT	-Ve	NT	NT	-Ve	NT	NT	NT	NT
		Viraemia	-Ve	-Ve	-Ve	-Ve	-Ve	-Ve	-Ve	-Ve	-Ve	-Ve	-Ve	-Ve	-Ve	-Ve	-Ve
		Skin nodules	-Ve	-Ve	-Ve	-Ve	-Ve	-Ve	-Ve	-Ve	-Ve	-Ve	-Ve	-Ve	-Ve	-Ve	-Ve
IVRI-1489	1X	Antibody titer	64	NT	NT	NT	NT	NT	NT	NT	NT	NT	NT	NT	NT	NT	256
		Lymphoproliferation	NS	NT	NT	NT	NT	NT	NT	NT	NT	NT	NT	NT	NT	NT	NT
		IFN-ϒ	-Ve	NT	NT	+Ve	NT	NT	NT	+Ve	NT	NT	-Ve	NT	NT	NT	NT
		Viraemia	-Ve	-Ve	-Ve	-Ve	-Ve	-Ve	-Ve	-Ve	-Ve	-Ve	-Ve	-Ve	-Ve	-Ve	-Ve
		Skin nodules	-Ve	-Ve	-Ve	-Ve	-Ve	-Ve	-Ve	-Ve	-Ve	-Ve	-Ve	-Ve	-Ve	-Ve	-Ve
IVRI-1524	1X	Antibody titer	32	NT	NT	NT	NT	NT	NT	NT	NT	NT	NT	NT	NT	NT	128
		Lymphoproliferation	NS	NT	NT	NT	NT	NT	NT	NT	NT	NT	NT	NT	NT	NT	NT
		IFN-ϒ	-Ve	NT	NT	+Ve	NT	NT	NT	-Ve	NT	NT	-Ve	NT	NT	NT	NT
		Viraemia	-Ve	-Ve	-Ve	-Ve	-Ve	-Ve	-Ve	-Ve	-Ve	-Ve	-Ve	-Ve	-Ve	-Ve	-Ve
		Skin nodules	-Ve	-Ve	-Ve	-Ve	-Ve	-Ve	-Ve	-Ve	-Ve	-Ve	-Ve	-Ve	-Ve	-Ve	-Ve
IVRI-1529	1X	Antibody titer	16	NT	NT	NT	NT	NT	NT	NT	NT	NT	NT	NT	NT	NT	64
		Lymphoproliferation	*	NT	NT	NT	NT	NT	NT	NT	NT	NT	NT	NT	NT	NT	NT
		IFN-ϒ	-Ve	NT	NT	+Ve	NT	NT	NT	+Ve	NT	NT	+Ve	NT	NT	NT	NT
		Viraemia	-Ve	-Ve	-Ve	-Ve	-Ve	-Ve	-Ve	-Ve	-Ve	-Ve	-Ve	-Ve	-Ve	-Ve	-Ve
		Skin nodules	-Ve	-Ve	-Ve	-Ve	-Ve	-Ve	-Ve	-Ve	-Ve	-Ve	-Ve	-Ve	-Ve	-Ve	-Ve
IVRI-745	1X	Antibody titer	16	NT	NT	NT	NT	NT	NT	NT	NT	NT	NT	NT	NT	NT	64
		Lymphoproliferation	NS	NT	NT	NT	NT	NT	NT	NT	NT	NT	NT	NT	NT	NT	NT
		IFN-ϒ	-Ve	NT	NT	-Ve	NT	NT	NT	-Ve	NT	NT	-Ve	NT	NT	NT	NT
		Viraemia	-Ve	-Ve	-Ve	-Ve	-Ve	-Ve	-Ve	-Ve	-Ve	-Ve	-Ve	-Ve	-Ve	-Ve	-Ve
		Skin nodules	-Ve	-Ve	-Ve	-Ve	-Ve	-Ve	-Ve	-Ve	-Ve	-Ve	-Ve	-Ve	-Ve	-Ve	-Ve
IVRI-1525	0	Antibody titer	<4	NT	NT	NT	NT	NT	NT	NT	NT	NT	NT	NT	NT	NT	32
		Lymphoproliferation	NA	NT	NT	NT	NT	NT	NT	NT	NT	NT	NT	NT	NT	NT	NT
		IFN-ϒ	-Ve	NT	NT	-Ve	NT	NT	NT	+Ve	NT	NT	-Ve	NT	NT	NT	NT
		Viraemia	-Ve	NT	NT	-Ve	NT	NT	NT	+Ve	NT	NT	+Ve	NA	NA	NA	-Ve
		Skin nodules	-Ve	-Ve	-Ve	-Ve	-Ve	-Ve	+Ve	+Ve	+Ve	+Ve	+Ve	+Ve	+Ve	+Ve	+Ve
IVRI-829	0	Antibody titer	<4	NT	NT	NT	NT	NT	NT	NT	NT	NT	NT	NT	NT	NT	32
		Lymphoproliferation	NA	NT	NT	NT	NT	NT	NT	NT	NT	NT	NT	NT	NT	NT	NT
		IFN-ϒ	-Ve	NT	NT	+Ve	NT	NT	NT	+Ve	NT	NT	-Ve	NT	NT	NT	NT
		Viraemia	NA	NA	NA	-Ve	NT	NT	NT	-Ve	NT	NT	+Ve	NT	NT	NT	-Ve
		Skin nodules	-Ve	-Ve	-Ve	-Ve	-Ve	-Ve	+Ve	+Ve	+Ve	+Ve	+Ve	+Ve	+Ve	+Ve	+Ve
IVRI-1492	0	Antibody titer	<4	NT	NT	NT	NT	NT	NT	NT	NT	NT	NT	NT	NT	NT	32
		Lymphoproliferation	NA	NT	NT	NT	NT	NT	NT	NT	NT	NT	NT	NT	NT	NT	NT
		IFN-ϒ	-Ve	NT	NT	+Ve	NT	NT	NT	-Ve	NT	NT	-Ve	NT	NT	NT	NT
		Viraemia	-Ve	NT	NT	-Ve	NT	NT	NT	+Ve	NT	NT	-Ve	NT	NT	NT	-Ve
		Skin nodules	-Ve	-Ve	-Ve	-Ve	-Ve	-Ve	+Ve	+Ve	+Ve	+Ve	+Ve	+Ve	+Ve	+Ve	+Ve

(−Ve): Negative; (+Ve: Positive); (NT): Not tested; (NA): Not applicable; (Ab)=Antibody titre; (NS): Non-significant difference; [Lymphoproliferation- ***P<0.001, **P<0.01, *P<0.05]; (IFN-ϒ): Detection of IFN-ϒ in serum; ($): Out of the five control animals, IVRI-835 and IVRI-1499, had anti-LSDV antibodies at the time of challenge (day 0 post-challenge), therefore these were not included in the analysis.

### Efficacy (Experimental trial)

**Figure 4.**
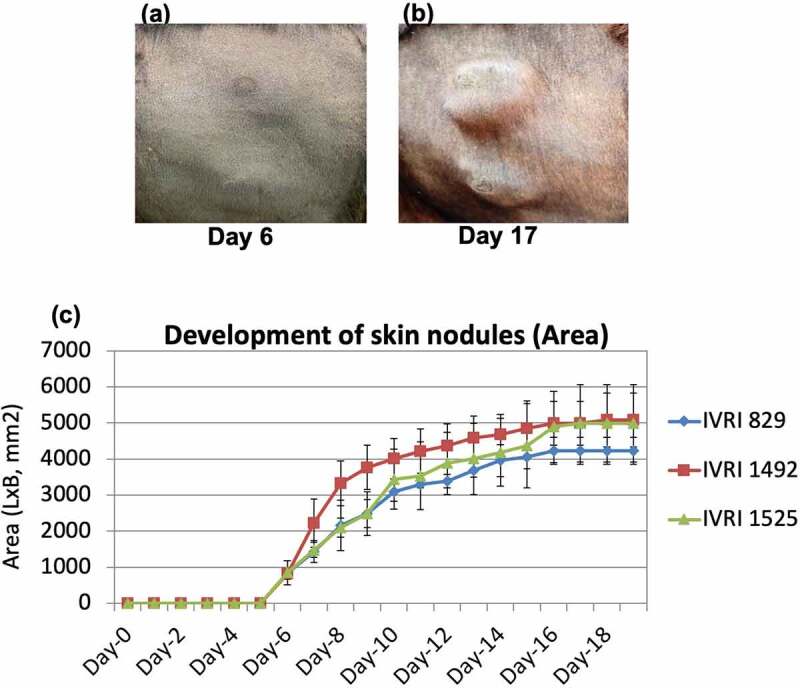
**Development and progression of skin nodules following challenge with virulent LSDV**. Animals were either unvaccinated or vaccinated with Lumpi-ProVac^Ind^. At day 30 pv, all the animals were challenged with virulent LSDV. Whereas vaccinated animals remained apparently healthy without showing any skin nodules, control (unvaccinated) animals developed the skin nodules following challenge. Primary swelling was seen at day 5–6 pc **(a)**, which progressively increased in size from ~600 mm^2^ (day 6 pc) to 5000 mm^2^ (day 16 pc) before becoming stable at day 17 pc **(b). The size of the developing skin nodule on various days post-challenge is also shown (c)**. IVRI 829, IVRI 1492 and IVRI 1525 are unvaccinated-challenged animals.

All the immunized animals (*n* = 10) along with the unvaccinated controls (*n* = 5) were challenged with the virulent LSDV on day 30 pv. All the control animals (*n* = 3 as 2 of the 5 control animals showed anti-LSDV antibodies in serum at day 0 pc and therefore were not included in the analysis) developed fever between 5 and 9 d pc which lasted for 1–4 d **(Supplementary Table S4)**. Localized skin nodules began appearing in all the unvaccinated control animals from day 5 to 6 pc (Table 4). The size of the skin nodules progressively increased from ~600 mm^2^ (day 6 pc) to 5000 mm^2^ (day 16 pc) before stabilizing at day 17 pc (Figure 4a-c). Skin nodules were observed at all four sites in all the control animals. Few generalized skin nodules could also be observed in the unvaccinated control animals, although large numbers of skin nodules, as seen in natural infection were not apparent. In addition, viraemia was also observed in all the unvaccinated-challenged animals between day 7–10 pc (Table 4). Besides, the unvaccinated control animals were also found to be anorectic and depressed at day 6–8 pc.

### Observations on the efficacy of Lumpi-ProVac^Ind^ in field animals

The LSD has become endemic in India. The outbreaks recorded have been very extensive with high morbidity and unusually high mortality. The field trials were initiated in clean (LSD free) herds during July–September 2022 and the animals were apparently healthy at the time of vaccination. Assuming that this is the first year of introduction of the disease (may be potentially free from maternal antibodies), we immunized all the animals including calves. Out of the 26,940 animals across 102 vaccinated farms, there has been no incidence of the disease till 11 January 2022 [except 14 animals (9 calves and 5 adults) across 5 farms wherein a mild disease was recorded] (Table 2), despite the fact that severe disease with significant mortality was observed in the nearby unvaccinated farms.

### Exposure of the vaccinated animals to virulent LSDV prior to development of a complete immunity

A complete immunity usually develops 3–4 weeks following LSD vaccination [30,31]. Since the disease was rampant and extensive outbreaks were being recorded in the surroundings farms/villages during the vaccine trials, vaccinated animals could have been exposed to the field/virulent virus. We observed the disease in 16 farms (in addition to 102 farms described above) at early times post-vaccination/during incubation period (Table 5). This was essentially due to insufficient immunity. In such cases, we were able to detect the field – but not vaccine the strain of LSDV from the skin nodules (Table 6) which suggested the association of field virus (not vaccine virus) in causation of the disease. The overall morbidity and case fatality rate in these 16 farms was observed to be 7.1% and 2.71%, respectively (Table 5).

**Table 5.** Morbidity and mortality in vaccinated animals that succumbed to natural infection before development of a complete immunity.

S. N.	House holding/farm	Total number of vaccinated animals	Number of animals succumbed to natural infection	Days after vaccination	Total deaths
1	LPV FT Raj-29	47	4	Day 3	0
2	LPV FT Raj-30	95	2	Day 4	0
3	LPV FT Raj-31	75	5	Day 14	0
4	LPV FT Raj-32	40	15	Day 10	0
5	LPV FT Raj-33	40	3	Day 10	0
6	LPV FT Raj-34	70	8	Day 3	3
7	LPV FT Raj-35	41	5	Day 10	0
8	LPV FT Raj-36	40	1	Day 4	0
9	LPV FT Raj-37	114	9	Day 4	0
10	LPV FT Raj-38	122	14	Day 3	2
11	LPV FT Raj-39	63	6	Day 6	0
12	LPV FT Raj-40	50	14	Day 2	1
13	LPV FT Raj-41	381	11	Day 7	0
14	LPV FT Raj-42	143	10	Day 10	0
15	LPV FT Raj-43	200	8	Day 3	0
16	LPV FT Raj-44	150	4	Day 2	0
	Total number of animals	1671	119		6
	**Morbidity/Case fatality rate**	**Morbidity= 7.1%**	**Case fatality rate= 2.71%**

**Table 6.** Differentiation of vaccine and field (virulent) strains of LSDV.

Sample ID	Nature of LSDV sample	qRT-PCR (cT values)	
		LSDV zdf4ln gene#	LSDV GPCR gene*
LSDV/P50	LSDV vaccine strain	12.562	16.37
LSDV/P0	LSDV field strain	12.464	Undetermined
Cell culture medium	Negative Control	Undetermined	Undetermined
2022–23/RJ/BKN/LSD/01	Field sample	15.059	Undetermined
2022–23/RJ/BKN/LSD/03	Field sample	14.934	Undetermined
2022–23/RJ/SRH/LSD/05	Field sample	14.974	Undetermined
2022–23/RJ/SRH/LSD/06	Field sample	15.204	Undetermined
2022–23/RJ/BDR/LSD/02	Field sample	18.445	Undetermined
2022–23/RJ/BDR/LSD/03	Field sample	18.59	Undetermined
RJ/SKR/LSD/Ssb-1	Field sample	18.868	Undetermined
RJ/SKR/LSD/Ssb-2	Field sample	18.961	Undetermined
22–23/RJ/LSD/SGNR/S-29	Field sample	15.992	Undetermined
22–23/RJ/LSD/SGNR/S-31	Field sample	15.97	Undetermined

#LSDV zdf4ln gene based qRT-PCR detects all LSDV strains but not other *Capripoxvirus* strains. *qRT-PCR is based on twelve base pair insertion in the GPCR gene in LSDV vaccine strains, thereby detects vaccine but not field strains.

### Vaccination of animals after appearance of the clinical disease

A limited number of animals in the selected farms that had developed the disease were also vaccinated. The overall case fatality rate in such instances was recorded to be 0.09% (Table 7).

**Table 7.** Case fatality rate in animals that were vaccinated after appearance of the clinical disease.

S. N.	Farm	Number of animals vaccinated (after infection)	Total deaths
1	LPV FT Raj-34	7	2
2	LPV FT Raj-45	10	3
3	LPV FT Raj-36	1	0
4	LPV FT Raj-33	3	0
5	LPV FT Raj-40	8	0
6	LPV FT Raj-38	14	2
7	LPV FT Raj-39	6	0
8	LPV FT Raj-40	14	1
9	LPV FT Raj-41	11	0
10	LPV FT Raj-43	8	0
11	LPV FT Raj-44	4	0
	Total number of animals	86	8
			**Case fatality rate=0.09%**

### Virus excretion in milk and semen

**Figure 5.**
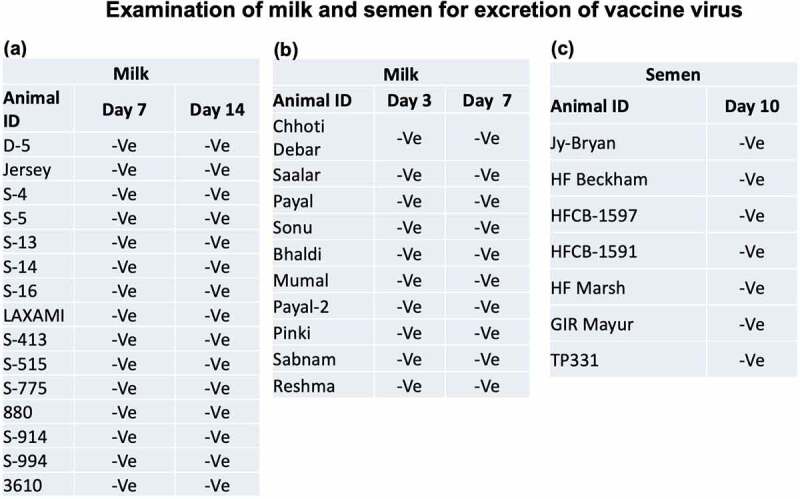
**Evaluation of the excretion of vaccine virus in milk and semen in vaccinated animals under field conditions**. Animals were vaccinated with Lumpi-ProVac^Ind^. Milk and semen samples were collected from the lactating cows and bulls respectively, and examined for the presence of LSDV genome by qRT-PCR. –Ve represents absence of LSDV genome.

We also evaluated the excretion of vaccine virus in milk of the vaccinated cows in the field. Paired milk samples, collected from day 3 to day 14 pv were found negative for LSDV genome by qRT-PCR (Figure 5a,b). Likewise, samples (*n* = 7) from bull semen collected on day 10 post-vaccination were also found negative for LSDV genome (Figure 5c).

### Cross neutralization of LSDV strains

Sera derived from LSDV vaccinated (LSDV/RCH/P50-vaccine strain) – or LSDV infected (LSDV/RCH/P2-virulent strain) cattle were subjected for their ability to neutralize different LSDV strains. These sera were able to equally neutralize various LSDV strains, irrespective of their history (2019 or 2022) or species of origin (camel or cattle) (Table 8).

**Table 8.** Neutralization of various LSDV strains by sera derived from LSDV/2019.

	Sera derived from LSDV 2019					
	*Vaccinated sera*^*#*^			*Infected sera*^*$*^		
*LSDV/Host species/History	IVRI-1489	IVRI-1524	IVRI-1533	IVRI-1525	IVRI-829	IVRI-1492
LSDV/Cattle/2019	64	32	32	32	32	32
LSDV/Cattle/2021	32	64	32	32	16	32
LSDV/Cattle/2022	64	32	32	32	32	32
LSDV/Camel/2022	64	32	64	16	32	32

#:Vaccinated sera were collected at day 30 post-vaccination. $: Infected sera were collected at day 14 post-infection. The values represent neutralizing antibody titre. *Details of the LSDV isolates can be found in materials and method section.

## Discussion

India is currently facing a deadly epidemic of LSD. Over 125,000 cattle have died and around 20 million cattle have been infected [1]. Capripoxviruses are genetically quite similar and antigenically indistinguishable; therefore all fall under a single serotype [32,33]. Due to the unavailability of a homologous LSD vaccine, the policy makers in India authorized the use of heterologous (GPV-based) vaccine to induce immunoprophylaxis against LSDV in cattle [14] as an emergency measure. However, heterologous vaccines provide only partial protection against LSDV in cattle and are not as efficacious as homologous LSDV-based vaccines, therefore necessitating a homologous LSD vaccine for vaccination in cattle [9,17–21,34,35]. This led us to develop a homologous live-attenuated LSD vaccine.

To develop a homologous live-attenuated LSD vaccine, a field virus from 2019 (Indian origin) was attenuated by continuous passages in Vero cells. As compared to the field/virulent strain (LSDV/P0), the whole-genome sequencing of the vaccine strain (LSDV/P50) revealed several mutations. The major mutations were observed in DNA-dependent RNA polymerase, Kelch-like protein, EEV membrane phospho-glycoprotein and CD47-like putative membrane protein, most of which have been reported in other *Capripoxvirus* vaccine strains as well [29]. A twelve bps insertion in the GPCR gene, which is considered as a signature mutation in vaccine strains [26] was observed even at P10 level and was maintained in subsequent passages in our study. In addition, vaccine strain had an 801 bp deletion in its inverted terminal repeat (ITR) region which has not been reported in any other vaccine/field strains. This major deletion could also be consistently detected at passage levels P40, P50, P60 and P70.

As compared to the classical Neethling LSDV strains which require over 100 passages for attenuation [36], KSGP strains require relatively low number of passages (~30) for attenuation [30]. Indian LSDV strains, including LSDV/2019/RCH which was used to prepare the vaccine is a NI-2490/Kenya/KSGP-like field strain (somewhere in between Neethling and KSGP LSDV strains) [5,37]. This, together with the similarities of LSDV/RCH/P50 with other *Capripoxvirus* vaccine strains tempted us to speculate about the potential attenuation of the P50 virus, and, therefore paving the way to conduct vaccine safety and efficacy studies in experimental calves.

The vaccine was well tolerated in calves with no local or systemic reaction or any other adverse effect on feed uptake, behaviour and general health status being observed. Also, the shedding of the vaccine virus was not detected in ocular, nasal and faecal swab from day 1 to day 30 pv. Further, viraemia (virus isolation from blood) could not be detected in any of the vaccinated animals, except that few of the animals had low copy numbers of viral genome in the blood at day 3 pv. Besides, various haematological and blood biochemical parameters were also found normal in the vaccinated animals. Taken together, experimental inoculation of the vaccine in cattle was found to be safe.

Upon challenge infection, all the unvaccinated (naïve) control animals developed localized skin nodules (onset at day 5–6 pc), besides developing viraemia and fever between day 7 and day 10 pc which lasted for 1–2 d. The unvaccinated control animals were also anorectic and depressed at day 7–8 pc. These data were comparable to previous studies on the experimental infection of LSD in cattle [33,38–42]. On the other hand, vaccinated-challenged animals completely resisted the development of skin nodules, fever and viraemia which strongly suggests that the LSDV/RCH/P50-based vaccine provides 100% protective efficacy against virulent LSDV.

Homologous live-attenuated vaccines are considered to be most immunogenic and efficacious. Neethling LSDV strain from South Africa has primarily been used as a homologous LSDV strain in most parts of the world [43]. Currently, at least eleven LSD vaccines have been described and are being used in different parts of the world to induce immunoprophylaxis against LSD in cattle [30]. Most of the homologous LSDV vaccines (Lumpyvax^TM^, Bovivax-LSD^TM^, LumpyShield-N^TM^ and MEVAC LSD) contain the well-known South African Neethling strain, despite their different passage/attenuation history [30]. Neethling strain-based vaccine was recently used to contain the disease in Balkan countries with great success [44]. The use of Kenyan sheep and goat pox (KSGP) virus strains O-240 and O-180, which were later confirmed as LSDV strains [13,45,46] has been limited to Egypt [47], Ethiopia [48] and Israel [4]. Besides, heterologous vaccines based on Yugoslavian RM65 sheep pox (SPP), Romanian SPP and Gorgan goat pox (GPT) strains have also been used in cattle against LSD [30]. Homologous live-attenuated LSD vaccines described above are known to cause adverse effects in cattle. This includes swelling at the site of vaccination or rarely generalized small-size skin nodules and a temporal reduction in the milk yield which is often referred to as “Neethling disease” or “Neethling response” [42,49,50]. In our study, none of the 10 vaccinated animals including 2 animals that received 10-times of field dose (10^4.5^ TCID_50_) developed any local or systemic reaction (nodule) following vaccination (experimental trial). In field conditions, only 0.018% of the animals (5 out of the 26,940 animals vaccinated) showed local site reaction (appeared at day 3 pv and subsided within 2 d) whereas generalized skin nodules were not reported in any of the vaccinated animals. In the previous studies under field conditions, the percentage of vaccinated animals exhibiting LSD-like nodules after vaccination was shown to vary from 0.09% to 12% [22,30,50,51]. Besides vaccine dose and immune status of the vaccinated animals, the nature of the vaccine strain used could be major factors for the Neethling response [30]. While harbouring most of the mutations reported in other *Capripoxvirus* vaccine strains, LSDV/2019/RCH/P50 (close to Kenyan LSDV strain) has a unique deletion of 801 nucleotides in its ITR region. This unique deletion mutation might be associated with extremely low levels of Neethling response in LSDV/RCH/P50-based vaccine, although further experimental proof will be essential.

A fever response is usually observed in animals following vaccination with Neethling strain [33,38–42]. In our study, rise in temperature following vaccination was recorded in a total of four animals viz; at day 12 to day 16 pv (four animals) and at day 1 to day 5 pv (two animals). We could not ascertain whether this rise in temperature is specific in response to vaccine or else due to other biotic or abiotic stress factors, because there was no specific pattern (onset and duration) of fever. Nevertheless, like in other existing LSDV/ other viral vaccines, fever for a few days in some of the vaccine recipients is a common phenomenon [30].

In natural infection, the infected animals may excrete virus in the milk [52] and semen [53]. Although there is no confirmed report on excretion of LSDV vaccine virus in the semen [53], a small percentage of the vaccinated cows may excrete virus in the milk [52,54]. In our study, we neither observed excretion of the vaccine virus in milk, nor in semen. This suggests that the milk from vaccinated animals (Lumpi-ProVac^Ind^) does not represent any public health hazard. Likewise, there is no risk of vertical transmission of vaccine virus via bull semen.

The seroconversion in our study was seen as early as day 18 pv (experimental trial). All the 10 vaccinated animals, except one, developed anti-LSDV antibodies (seroconversion rate 90%) on the day 30 pv. In the field studies, seroconversion rate was 85.18% (*n* = 648) at day 30 pv. Seroconversion rate following LSD vaccination is highly variable (40–80%) [55–58]. Since LSD has become endemic in India and extensive outbreaks were being recorded at the time of the vaccine trials, it is likely that the high seroconversion rate is presumably due to exposure to natural infection, post-vaccination. This essentially necessitates the development of a test system to differentiate among the antibodies generated due to infection or vaccination.

Although the under trial animals were tested negative for antibodies against capripoxviruses before start of the safety and efficacy trials, but one of the vaccinated animal (IVRI 1462) and two (IVRI-835 and IVRI-1499) of the five control animals had anti-LSDV antibodies at the time of vaccination (day 0 pv), and at challenge (day 0 pc) infection respectively. Unvaccinated control animals with pre-existing antibodies (unknown history of LSDV exposure) at the time of challenge infection were completely protected upon virulent challenge. This suggests that they were essentially exposed to LSDV in between screening (for seronegativity) and the time of challenge. Interestingly, these animals also exhibited an IFY-ϒ response in serum. Whether increased IFY-ϒ in these animals was due to LSDV exposure or due to cryptic infections [59] could not be ascertained.

Lymphoproliferation and levels of IFN-ϒ were estimated as a measure of cell-mediated immunity. Significant cell proliferation was observed in 60% of the vaccinated calves at day 30 pv. In agreement with the previous studies [55,56,60], the peak response of IFN-ϒ was observed at day 3 post LSDV exposure (irrespective of vaccination/infection), the highest being in vaccinated-challenged (100%) than in unvaccinated-challenged (66%) or merely vaccinated (50%) animals.

Although most of vaccinated animals in our study developed both antibody- and cell-mediated immune response, some animals did not show measurable amount of antibody and/or cell-mediated immune response which may be due to the fact that timing and magnitude of immune response to LSDV may vary from animal-to-animal [61]. However, all the vaccinated animals resisted the challenge with the virulent LSDV. With the reasons unknown, protection from virulent LSDV in the absence of detectable anti-LSDV neutralizing antibodies has also been reported by other workers [17,42,53,62]. While antibody response following vaccination of cattle with LSDV is variable (40–80%), the antibodies may be undetectable following administration of SPV vaccines [16,63]. This may partly be attributed to the nature of some cattle breeds or the type of vaccine used [64,65]. In a study by Kafafy *et al*., no serological response was induced in cattle vaccinated with Romania SP strain while a trivalent Capripox vaccine (made up of SP Romania, GTPV Held and KSGP 0180) induced antibodies in 66% of vaccinated animals [63].

In our study, the levels of IFN-ϒ in serum increased from its basal level (at day 0 pv) to attain peak titres at day 3 pv, before declining at day 7 and coming back to basal level after day 10 pv. This suggested the induction of cell-mediated immune response by the vaccine. As compared to day 0 pc (day 30 pv) where IFN-ϒ was at its basal level, 100% of the vaccinated animals showed IFN-ϒ response at day 3 pc, suggesting that all the vaccinated animals had developed vaccine-induced cell-mediated immune response prior to the challenge infection. This was further confirmed by lymphoproliferative response of PBMCs following stimulation with inactivated LSDV antigen. However, a significant lymphoproliferative response was detected in ~60% of the vaccinated animals at day 30 pv. The response of cattle inoculated with LSD vaccine substantially varies showing a different timing and magnitude of response across different test system being employed viz; Interferon Gamma Release Assay (IGRA), intracellular cytokine staining (ICS) and Enzyme-linked immunosorbent spot (ELISpot) assay [61]. Nevertheless, the kinetics and magnitude of cell-mediated immune response against LSDV, and the role it plays in protection against disease, is very poorly understood [10]. This needs further investigation.

Although none of the live-attenuated capripoxvirus vaccines have been claimed to provide total protection, however, the spread of LSD has been successfully controlled using Neethling vaccines at high levels of vaccine coverage [3]. LSD vaccines are widely used in Africa, although vaccine breakdown and reinfection of vaccinated animals have been reported [66]. The heterogeneity of viruses used in vaccine, use of over attenuated virus strain and inappropriate production process (quality) of the vaccine may lead to vaccine failure [17,18,30,38]. However, with a vaccine effectiveness of up to 97%, Neethling vaccine was recently shown to be highly effective in controlling the LSD epidemic in six Balkan countries [44]. Although our analysis included the observations for a maximum of 4–6 months post-vaccination, the efficacy of Lumpi-ProVac^Ind^ was determined to be 99.94% [out of 26,940 animals, only 14 animals (0.05%) developed the disease after completion of 21 d of vaccination]. The numbers of calves included for vaccination in our study were <5%, however, the majority of the total cases of vaccine ineffectiveness in our study were observed in calves (52.52%). The poor efficacy of the vaccine in these selected calves might be attributed to interference of vaccination by maternal antibodies [22,67]. However, this needs further investigation.

The field trials were initiated in clean disease-free herds. However, since the disease was widely prevalent, some of the animals exhibited symptoms of LSD early post-vaccination, which was presumably due to encountering the natural infection and was confirmed by differentiating the field virus with the vaccine virus by PCR. Since full protective immunity is believed to develop only after 3–4 weeks post-vaccination [30,31], the natural disease is expected during the period that bridges between vaccination and development of complete immunity. All such cases were observed in Rajasthan which was the most badly affected state. In such instances, the overall morbidity and case fatality rate in the vaccinated animals was 7.1% and 2.71%, respectively (Table 4). This was significantly low as compared to the overall morbidity and case fatality rate (11.28% and 4.8% respectively) in the Rajasthan state where goatpox vaccine was practiced [68]. This suggests a beneficial effect of Lumpi-ProVac^Ind^. Therefore, our findings appear to suggest the use of LSD vaccine during epidemics, irrespective of the history of the animals in close contact with the infected animals. However, there are conflicting reports on the outcome of the disease in vaccinated animals that succumb to natural infection at early times post-vaccination. A study by Ayelet suggests high morbidity in local breed but no significant effect in cross breeds during the outbreak in the vaccinated animals [48]. Study by Abutrabush suggested highest morbidity and mortality in the order of nonvaccinated farms >vaccinated farms after infection >vaccinated farms [18]. It is speculated that while the virulent virus is preparing for tissue damage during disease progression, some immunity elicited by the vaccine virus could neutralise the virulent virus, thus slowing the disease process. However, this aspect of pathogenesis and protection mechanism in infected and vaccinated animals needs experimental proof and in-depth investigation.

Likewise, the average case fatality rate in the animals that were vaccinated after appearance of the clinical disease was low (0.09%) as compared to the overall case fatality rate (4.8%) in the state. This also appears to suggest that in most instances, vaccination has a beneficial effect, at least reducing the disease severity. However, the case fatality rate, which varied from 0% to 30% (Table 7), also appears to suggest that in certain instances, vaccination of infected animals may have an adverse impact on disease severity. The beneficial or adverse impact may depend on the time of vaccinating infected animals, besides coinfection of other microbial agents [59]. This needs further investigation.

A cross protection has been established among various capripoxviruses in general, and among the LSDV strains in particular [1]. However, due to the unusual high mortality involved in the recent LSDV outbreak in the Indian subcontinent, concerns were raised about the protective efficacy of the vaccine. The sera derived from LSDV/2019 (candidate vaccine virus) were able to equally neutralize LSDV/2019, LSDV/2021, and LSDV/2022. This, together with high efficacy of Lumpi-ProVac^Ind^ in the field (against LSDV/2022) suggests that the vaccine can be conveniently employed for control and eradication of LSD against multiple LSDV strains.

## Conclusion

The safety profile of Lumpi-ProVac^Ind^ is very high (minimal or no Neethling response) as compared to the other existing live-attenuated vaccines. It was found to be highly efficacious against LSD in experimental and field trials and could prove to be a better option for the control and eradication of LSD in India as well as other affected countries.

## Supplementary Material

Supplemental Material

## Data Availability

The data that support the findings of this study are openly available in the concerned repositories. LSDV field strains used in this study were isolated by our group and deposited at National Centre for Veterinary Type Cultures, Hisar, India (repository of animal microbes; http://ncvtc.org.in/) with Accession Numbers VTCCAVA288 (LSDV/cattle/2019), VTCCAVA 321 (LSDV/cattle/2021), VTCCAVA 370 (LSDV/cattle/2022) and VTCCAVA 371 (LSDV/camel/2022). The complete nucleotide sequences of field (LSDV/2019/RCH/P0) and vaccine strain (LSDV/2019/RCH/P50) is available with GenBank Accession Number of MW883897.1 and OK422494.1, respectively.
