# Supplementary material for: Evaluation of the safety, immunogenicity and efficacy of a new live-attenuated lumpy skin disease vaccine in India
Source: Virulence. 2023 Mar 19;14(1):2190647. doi: 10.1080/21505594.2023.2190647 (PMC10038050; doi:10.1080/21505594.2023.2190647)
Supplement: Supplemental Material [file KVIR_A_2190647_SM6133.docx]

**Supplementary Tables and Figures**

| **Supplementary table 1: Body temperature of calves following vaccination (Experimental trial)** | | | | | | | | | | | | | | | | | | | |
| --- | --- | --- | --- | --- | --- | --- | --- | --- | --- | --- | --- | --- | --- | --- | --- | --- | --- | --- | --- |
| **Dose** | **Identity** | **Day 0** | **Day1** | **Day2** | **Day3** | **Day4** | **Day5** | **Day6** | **Day7** | **Day8** | **Day9** | **Day10** | **Day11** | **Day12** | **Day13** | **Day14** | **Day15** | **Day16** | **Day17** |
| **10X dose** | **IVRI-1519** | 102.4 | 102.6 | 101.5 | 102.1 | 101 | 102.8 | 100 | 101.7 | 101.9 | 100.9 | 100.3 | 101 | 103.2 | 103 | **103.1** | **103** | **103** | 100.2 |
|  | **IVRI-1552** | 101.8 | 102.1 | 101.7 | 102.6 | 101.6 | 102.3 | 101.8 | 100.4 | 102.3 | 102.1 | 101 | 101.5 | 101.4 | 102.2 | 102.1 | 102.1 | 102.2 | 100.5 |
| **1X dose** | **IVRI-1550** | **103.1** | 101.6 | 101.6 | 101.7 | 101.3 | 102.4 | 102.1 | 101.4 | 102.1 | 102 | 101.3 | 101.3 | 100.9 | 101.1 | 100.1 | 101.7 | 101.6 | 100.6 |
|  | **IVRI-1541** | 102.2 | 102.3 | 101.7 | **103.1** | 101.9 | 102.4 | 102.2 | 101.8 | 102.4 | 100.2 | 101.4 | 102 | 102.5 | 102.4 | **103.1** | **103.3** | **103.2** | 101.9 |
|  | **IVRI-1533** | 101.8 | 101.7 | 101.5 | 102.2 | 101 | 102.5 | 100.1 | 100.9 | 102.2 | 101.4 | 100.5 | 101 | 101.7 | 101.7 | 101.4 | 102.2 | 102.3 | 99.8 |
|  | **IVRI-1462** | 100.9 | 101.4 | 101.2 | 100.8 | 100.9 | 101.2 | 101.1 | 100.6 | 101.3 | 101.9 | 100.8 | 100.7 | 102.1 | 101.6 | 101.9 | 102.2 | 102.1 | 100.1 |
|  | **IVRI-1489** | 100.6 | 102.6 | 102.3 | 102.4 | 100.9 | 101.7 | 99.5 | 100.7 | 100.6 | 101.4 | 99.9 | 101.4 | 101.3 | 101.7 | 100.5 | 101.7 | 101.6 | 100.1 |
|  | **IVRI-1524** | 101.3 | 102 | 101.5 | 101.4 | 100.9 | 102.2 | 100.3 | 100.7 | 101.5 | 100.5 | 100.2 | 101.2 | 100.9 | 101.8 | 101.6 | **103.1** | **103.2** | 99.2 |
|  | **IVRI-1529** | 101.9 | **103** | 102.4 | **103** | 101.8 | **103.1** | 102.3 | 101.3 | 102.6 | 101.7 | 100.9 | 102.4 | **103.7** | **103.4** | **104** | **103.2** | **103.1** | 100.9 |
|  | **IVRI-745** | 101.2 | 101.5 | 101 | 101.7 | 99.9 | 101.7 | 100.5 | 100.4 | 100.6 | 100.3 | 99.8 | 101.1 | 100.9 | 100.4 | 100.7 | 101 | 101.1 | 100.2 |

| **Supplementary table 2: Body temperature of cattle following vaccination with Lumpi-ProVac^Ind^ (field trial)** | | | | | | | | | | | | | | | | | | |
| --- | --- | --- | --- | --- | --- | --- | --- | --- | --- | --- | --- | --- | --- | --- | --- | --- | --- | --- |
| **Animal ID** | | **Day 1** | | **Day 2** | | | **Day 3** | | **Day 4** | | | **Day 5** | | | **Day 6** | | **Day 7** |  |
| **Cow** | | **20-08-2022** | | **21-08-2022** | | | **22-08-2022** | | **23-08-2022** | | | **24-08-2022** | | | **25-08-2022** | | **27-08-2022** |  |
| **319** | | 102.8 | | 101.2 | | | 101.8 | | 100.2 | | | 101.4 | | | 100.8 | | 101.2 |  |
| **363** | | 99.8 | | 102 | | | 101.4 | | 101.2 | | | 100.8 | | | 101.2 | | 102.4 |  |
| **419** | | 102 | | 101.4 | | | 103 | | 100.8 | | | 101.6 | | | 101.2 | | 100.8 |  |
| **424** | | 102.6 | | 103 | | | 102.2 | | 101.6 | | | 101 | | | 100.8 | | 102.2 |  |
| **471** | | 101.6 | | 100.8 | | | 100.2 | | 100 | | | 102.2 | | | 101 | | 101.6 |  |
| **460** | | 100 | | 101.2 | | | 101.6 | | 101.2 | | | 101.6 | | | 99.8 | | 101.4 |  |
| **467** | | 103.2 | | 102.4 | | | 103 | | 101.8 | | | 100.8 | | | 101.2 | | 100.8 |  |
| **454** | | 102.4 | | 101.8 | | | 101.4 | | 102 | | | 101.2 | | | 101.6 | | 101.2 |  |
| **490** | | 100.8 | | 102.4 | | | 103.2 | | 101.4 | | | 101.6 | | | 100.2 | | 102.8 |  |
| **526** | | 100.2 | | 101.8 | | | 102.2 | | 102.8 | | | 101.2 | | | 100.8 | | 101.6 |  |
|  | |  | |  | | |  | |  | | |  | | |  | |  |  |
| **Heifer** | | **20-08-2022** | | **21-08-2022** | | | **22-08-2022** | | **23-08-2022** | | | **24-08-2022** | | | **25-08-2022** | | **27-08-2022** |  |
| **497** | | 101.2 | | 101.6 | | | 102.6 | | 100.2 | | | 101.2 | | | 101.2 | | 102.4 |  |
| **513** | | 101.2 | | 100.8 | | | 101.8 | | 100.8 | | | 101.4 | | | 101.6 | | 100.8 |  |
| **524** | | 101.8 | | 101.6 | | | 100.2 | | 99.6 | | | 100.6 | | | 102.2 | | 101.8 |  |
| **532** | | 103 | | 101.8 | | | 102.6 | | 101.2 | | | 101.2 | | | 101.6 | | 102.4 |  |
| **547** | | 100.4 | | 100.2 | | | 100.8 | | 100.2 | | | 101.8 | | | 103.2 | | 101.2 |  |
|  | |  | |  | | |  | |  | | |  | | |  | |  |  |
| **Cow** | | **23-08-2022** | | **24-08-2022** | | | **25-08-2022** | | **27-08-2022** | | | **28-08-2022** | | | **29-08-2022** | | **30-08-2022** |  |
| **504** | | 101.8 | | 100.6 | | | 101.2 | | 102.2 | | | 101.8 | | | 101.2 | | 101.4 |  |
| **502** | | 102.6 | | 101.2 | | | 101.6 | | 101.2 | | | 100.6 | | | 100.4 | | 101.2 |  |
| **463** | | 103.2 | | 102.4 | | | 100.4 | | 102.6 | | | 101.2 | | | 101.6 | | 100.8 |  |
| **438** | | 101.2 | | 100.8 | | | 102.2 | | 101.4 | | | 101.8 | | | 102 | | 101.4 |  |
| **428** | | 101.2 | | 101.6 | | | 101.8 | | 103.2 | | | 102.2 | | | 102 | | 102.2 |  |
|  | |  | |  | | |  | |  | | |  | | |  | |  |  |
| **Male** | | **23-08-2022** | | **24-08-2022** | | | **25-08-2022** | | **27-08-2022** | | | **28-08-2022** | | | **29-08-2022** | | **30-08-2022** |  |
| **585** | | 102 | | 103.2 | | | 101.2 | | 102.2 | | | 101.8 | | | 101.4 | | 101.6 |  |
| **586** | | 101.2 | | 102.8 | | | 101.8 | | 100.6 | | | 101.2 | | | 102.6 | | 100.8 |  |
| **540** | | 102.8 | | 101 | | | 102.2 | | 101.2 | | | 102.2 | | | 101.2 | | 101.4 |  |
| **581** | | 103.2 | | 102.2 | | | 101 | | 102.4 | | | 102.8 | | | 101.2 | | 102.2 |  |
| **579** | | 103 | | 103.4 | | | 102.6 | | 101.8 | | | 101.2 | | | 102.4 | | 100.6 |  |
| Cattle farm at Krishi Vigyan Kendra, ICAR-Central Arid Zone Research Institute, Jodhpur, India | | | | | | | | | | | | | | | | | |  |
| **Supplementary table 3: Body temperature of buffaloes following vaccination with Lumpi-ProVac^Ind^ (Field trial)** | | | | | | | | | | | | | | | |  |  |  |
|  |  |  |  |  |  |  |  |  |  |  |  |  |  |  |  |  |  |  |
| **Animal ID** | **Day 0** | | **Day 1** | | **Day 2** | **Day 3** | | **Day 4** | | **Day 5** | **Day 6** | | **Day 7** | **Day 8** | |  |  |  |
| 4494 | 101.5 | | 101.4 | | 100.5 | 100.4 | | 100.9 | | 101.2 | 101 | | 100.7 | 101.2 | |  |  |  |
| 4501 | 102.1 | | 100.9 | | 101 | 100.9 | | 101 | | 101.2 | 100.5 | | 101 | 102 | |  |  |  |
| 4537 | 100.9 | | 101.6 | | 100.8 | 101.0 | | 101.3 | | 100.6 | 101.2 | | 99.9 | 100.7 | |  |  |  |
| 4549 | 101.6 | | 101.5 | | 101.0 | 99.6 | | 100.5 | | 101.0 | 99.9 | | 101.2 | 101.1 | |  |  |  |
| 4582 | 102.0 | | 101.3 | | 101.0 | 100.9 | | 101.4 | | 101.0 | 102.1 | | 99.8 | 101.2 | |  |  |  |
| 4613 | 101.4 | | 102.1 | | 100.6 | 99.6 | | 101.9 | | 100.9 | 101.1 | | 100.8 | 100.9 | |  |  |  |
| 4616 | 100.5 | | 101.2 | | 100.0 | 100.3 | | 101.5 | | 100.8 | 101.7 | | 100.1 | 101.8 | |  |  |  |
| 4625 | 101.2 | | 101.1 | | 100.3 | 100.1 | | 102.1 | | 100.2 | 101.7 | | 102.1 | 101.7 | |  |  |  |
| 4632 | 99.9 | | 101.5 | | 101.0 | 100.7 | | 100.9 | | 100.8 | 101.0 | | 100.2 | **102.5** | |  |  |  |
| 4676 | 102.1 | | 102.4 | | 100.8 | 99.6 | | 101.6 | | 99.7 | 100.9 | | 102.1 | 102.2 | |  |  |  |
| 4702 | 101.8 | | 101.6 | | 100.6 | 100.1 | | 102.0 | | 101.4 | 102.5 | | 101.7 | 99.7 | |  |  |  |
| 4735 | 102.0 | | 101.3 | | 100.3 | 100.2 | | 101.4 | | 101.2 | 100.9 | | 100.8 | 101.7 | |  |  |  |
| 4741 | 100.9 | | 102.2 | | 100.7 | 99.4 | | 101.8 | | 100.6 | 99.8 | | 99.2 | 99.7 | |  |  |  |
| 4745 | 99.8 | | 101.8 | | 99.8 | 100.0 | | 102.0 | | 100.9 | 102.1 | | 101.7 | 100.8 | |  |  |  |
| 4749 | 100.8 | | 100.0 | | 100.3 | 100.1 | | 100.9 | | 100.5 | 100.9 | | 101.1 | 100.6 | |  |  |  |
| 4771 | 100.1 | | 102.4 | | 100.6 | 100.5 | | 101.6 | | 101.2 | 99.7 | | 101.7 | 99.8 | |  |  |  |
| 4876 | 101.6 | | 100.8 | | 100.4 | 100.5 | | 101.8 | | 102.0 | 100.1 | | 101.5 | 101.5 | |  |  |  |
| 4885 | 102.1 | | 101.4 | | 99.8 | 100.0 | | 101.1 | | 101.0 | 100.9 | | 99.7 | 101.3 | |  |  |  |
| Surti Buffallo farm, Rajasthan University of Veterinary and Animal Sciences (RAJUVAS), Navaina, Udaipur (Rajasthan), India | | | | | | | | | | | | | | | |  |  |  |

| **Supplementary Table 4: Body temperature of vaccinated and unvaccinated animals following challenge with virulent LSDV (Experimental trial)** | | | | | | | | | | | | | | | | | |
| --- | --- | --- | --- | --- | --- | --- | --- | --- | --- | --- | --- | --- | --- | --- | --- | --- | --- |
|  | **Dose** | **Animal ID** | **D-0** | **Day-1** | **Day-2** | **Day-3** | **Day-4** | **Day-5** | **Day-6** | **Day-7** | **Day-8** | **Day-9** | **Day-10** | **Day-11** | **Day-12** | **Day-13** | **Day-14** |
| Vaccinated Animals | 10X | IVRI-1519 | 102.1 | 100.7 | 101.8 | 101.1 | 101.0 | 100.3 | 100.3 | 100.0 | 100.3 | 100.6 | 100.0 | 100.0 | 99.0 | 100.9 | 100.6 |
|  |  | IVRI-1552 | 103.4 | 102.1 | 100.7 | 101.9 | 100.7 | 101.5 | 101.6 | 100.9 | 101.7 | 100.7 | 100.1 | 100.0 | 100.0 | 100.0 | 100.1 |
|  | 1X field dose | IVRI-1550 | 102.2 | 101.0 | 100.6 | 101.4 | 99.9 | 100.7 | 100.4 | 100.0 | 101.1 | 101.4 | 100.0 | 100.0 | 100.1 | 100.5 | 100.5 |
|  |  | IVRI-1541 | 102.2 | 102.1 | 101.6 | 101.9 | 101.3 | 101.7 | 101.1 | 100.7 | 101.5 | 102.7 | 101.4 | 100.4 | 101.3 | 101.0 | 100.8 |
|  |  | IVRI-1533 | 101.4 | 100.4 | 100.0 | 101.1 | 100.2 | 100.5 | 100.8 | 100.0 | 102.0 | 99.8 | 100.2 | 100.3 | 100.0 | 100.0 | 100.0 |
|  |  | IVRI-1462 | 101.7 | 101.8 | 100.7 | 101.6 | 101.1 | 101.3 | 100.8 | 100.1 | 100.8 | **103.3** | 101.7 | 101.3 | 100.0 | 99.1 | 100.0 |
|  |  | IVRI-1489 | 101.8 | 100.5 | 99.8 | 100.0 | 100.1 | 100.8 | 100.9 | 100.0 | 102.5 | 100.0 | 100.0 | 100.1 | 100.1 | 100.7 | 100.3 |
|  |  | IVRI-1524 | 101.7 | 100.9 | 99.8 | 101.1 | 101.5 | 100.6 | 100.9 | 100.2 | 100.0 | 101.0 | 100.0 | 100.1 | 100.2 | 100.8 | 100.6 |
|  |  | IVRI-1529 | 102.2 | 101.9 | 101.4 | 102.2 | 101.3 | 101.7 | 100.1 | 101.3 | 101.7 | 101.1 | 100.9 | 100.1 | 100.2 | 101.7 | 100.9 |
|  |  | IVRI-745 | 101.6 | 100.6 | 100.7 | 100.6 | 101.7 | 102.0 | 101.5 | 100.0 | 100.8 | 100.6 | 100.1 | 100.0 | 100.0 | 100.0 | 100.1 |
| Control animals | NA | IVRI-835***** | 101.1 | 99.4 | 100.3 | 100.7 | 100.4 | 101.1 | 100.8 | 100.0 | 100.2 | 100.9 | 100.1 | 100.0 | 100.3 | 100.3 | 100.6 |
|  |  | IVRI-1525 | 100.9 | 100.5 | 100.6 | 100.5 | 100.4 | **103.1** | **104.2** | **103.2** | 102.1 | **103.3** | 102.9 | 101.8 | 102.3 | 101.2 | 100.8 |
|  |  | IVRI-1499***** | 100.9 | 102.6 | 100.2 | 100.1 | 101.4 | 101.7 | 101.3 | 100.6 | 100.6 | 102.7 | 100.4 | 100.9 | 100.4 | 100.8 | 100.6 |
|  |  | IVRI-829 | 101.3 | 100.6 | 99.7 | 101.1 | 102.3 | 101.1 | 100.8 | 100.0 | **104.2** | 100.0 | 103.1 | 100.4 | 100.2 | 100.0 | 100.0 |
|  |  | IVRI-1492 | 101.5 | 100.1 | 100.8 | 100.9 | 100.5 | 101.4 | 101.8 | **103.3** | **104.3** | **103.7** | 101.4 | 101.5 | 100.5 | 101.3 | 101.0 |
| IVRI-835* and IVRI-1499* had anti-LSDV antibodies at the time of challenge (Day 0 post-challenge), therefore not included in the analysis | | | | | | | | | | | | | | | | | |


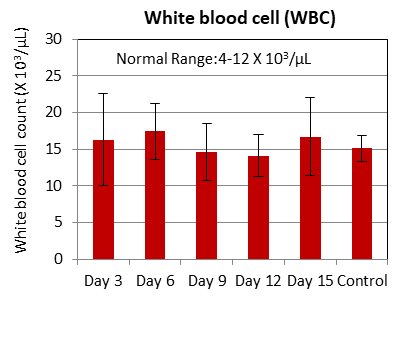

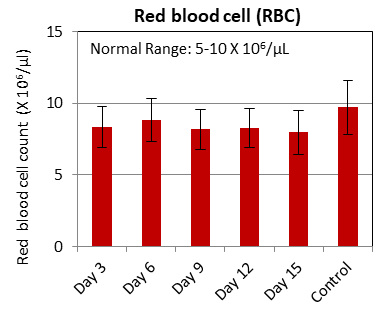

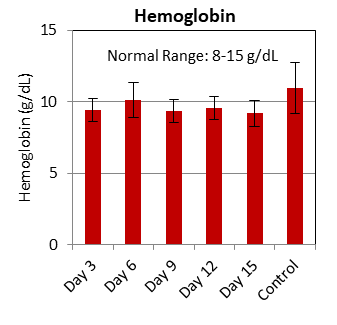

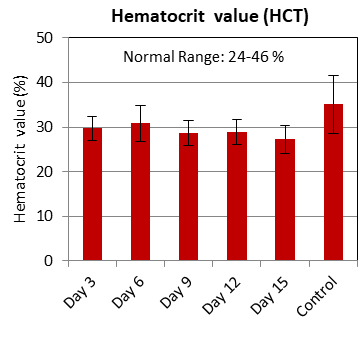

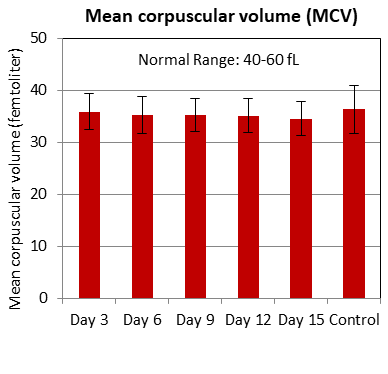

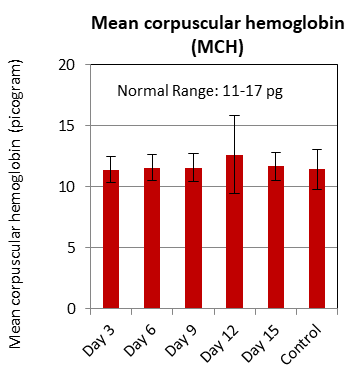

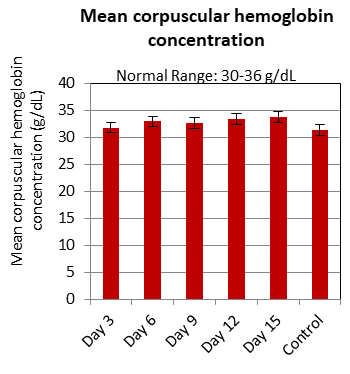

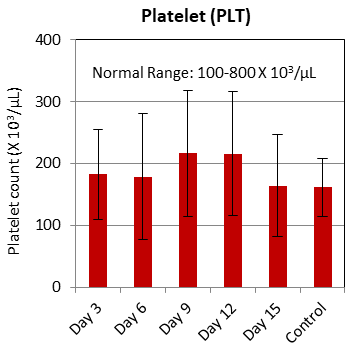

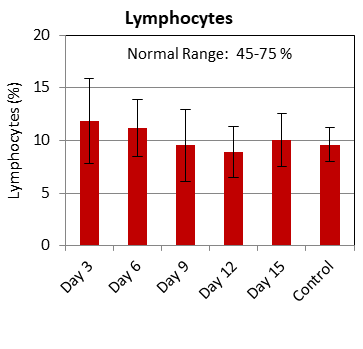

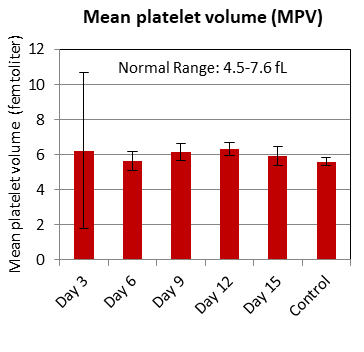

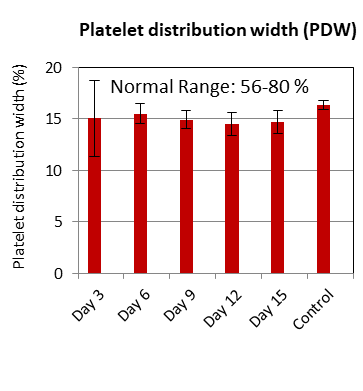

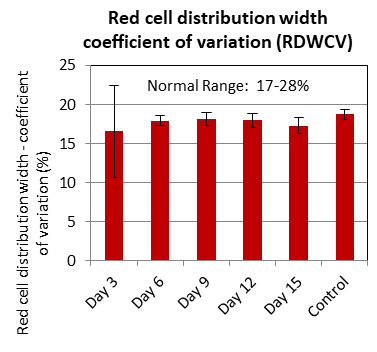


**Supplementary Fig S1. Various blood parameters in vaccinated- and unvaccinated animals at various times after vaccination**

**Supplementary Fig S2: Various blood biochemical parameters in vaccinated- and unvaccinated animals at various times after vaccination**


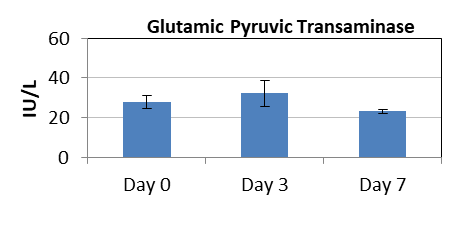


(Range 35.5-57.4 IU/L)


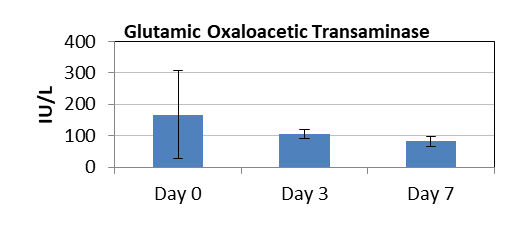


(Range 122.0-163.1 IU/L)


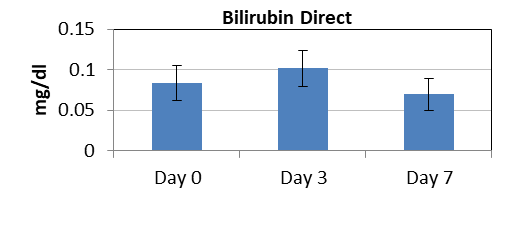


(Range 0.001-0.02 mg/dl)


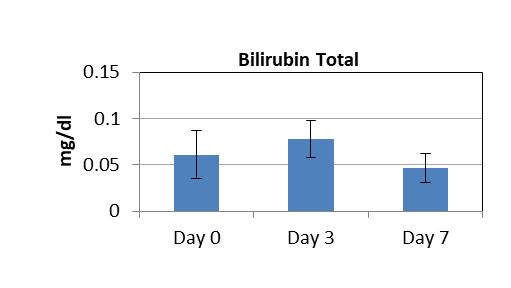


(Range 0.01-0.11 mg/dl)


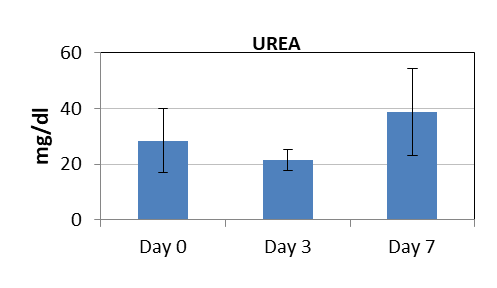


(Range 39.6-77.5 mg/dl)


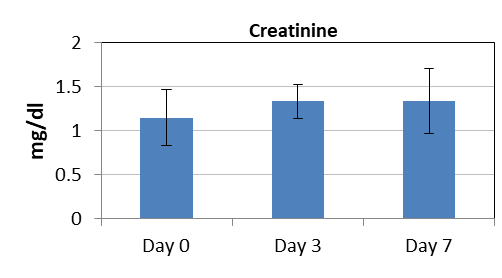


(Range 0.0-1.5 mg/dl)


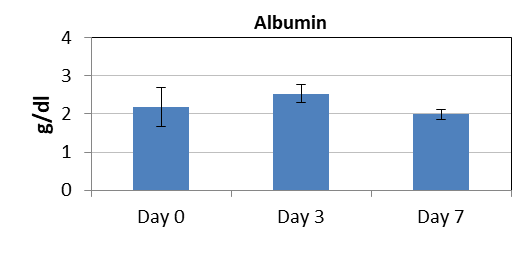

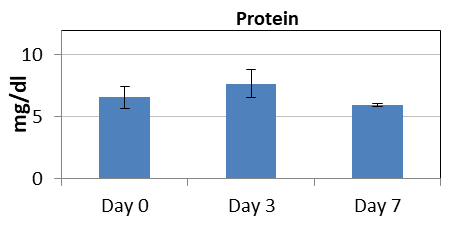

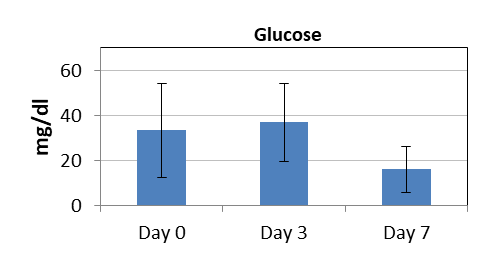


(Range 45-75 mg/dl)

(Range 3.2-3.6 g/dl)

(Range 10-15 mg/dl)


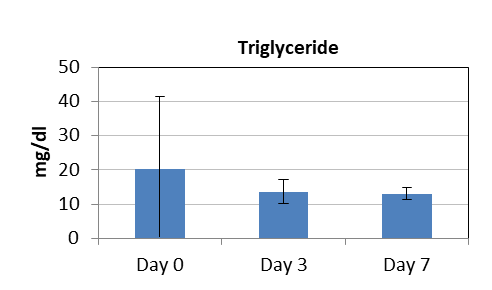

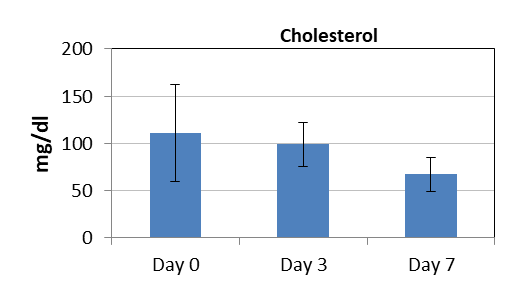

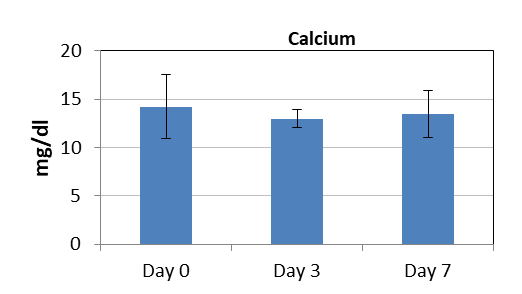


(Range <150 mg/dl)

(Range 36.0-90.1 mg/dl)

(Range 2.81-10.91 mg/dl)
